# Trends in Antarctic Ice Sheet Elevation and Mass

**DOI:** 10.1029/2019GL082182

**Published:** 2019-07-24

**Authors:** Andrew Shepherd, Lin Gilbert, Alan S. Muir, Hannes Konrad, Malcolm McMillan, Thomas Slater, Kate H. Briggs, Aud V. Sundal, Anna E. Hogg, Marcus E. Engdahl

**Affiliations:** ^1^ Centre for Polar Observation and Modelling, School of Earth and Environment University of Leeds Leeds UK; ^2^ Mullard Space Science Laboratory, Department of Space & Climate Physics University College London London UK; ^3^ Centre for Polar Observation and Modelling, Department of Earth Sciences University College London London UK; ^4^ Now at Deutscher Wetterdienst Offenbach Germany; ^5^ Now at Centre for Polar Observation & Modelling, Centre of Excellence in Environmental Data Science Lancaster University Lancaster UK; ^6^ ESA‐ESRIN, Via Galileo Galilei Frascati Italy

**Keywords:** climate, Antarctica, satellite, altimetry, imbalance, sea level

## Abstract

Fluctuations in Antarctic Ice Sheet elevation and mass occur over a variety of time scales, owing to changes in snowfall and ice flow. Here we disentangle these signals by combining 25 years of satellite radar altimeter observations and a regional climate model. From these measurements, patterns of change that are strongly associated with glaciological events emerge. While the majority of the ice sheet has remained stable, 24% of West Antarctica is now in a state of dynamical imbalance. Thinning of the Pine Island and Thwaites glacier basins reaches 122 m in places, and their rates of ice loss are now five times greater than at the start of our survey. By partitioning elevation changes into areas of snow and ice variability, we estimate that East and West Antarctica have contributed −1.1 ± 0.4 and +5.7 ± 0.8 mm to global sea level between 1992 and 2017.

## Key Points

- We combine satellite radar altimetry and a regional climate model to separate changes in Antarctic elevation into snow and ice contributions
- Between 1992 and 2017, ice thinning has grown in extent to include 24% of West Antarctica and reaches 122 m in places
- Ice losses from Pine Island and Thwaites Glaciers have risen fivefold; East and West Antarctica have contributed 4.6 ± 1.2 mm to sea level

## 1 Introduction

Satellite observations have transformed our knowledge of the Antarctic Ice Sheet (AIS) (Vaughan et al., 2013) and are now an important observational constraint for numerical simulations of its response to future climate change (Shepherd & Nowicki, 2017). Today, three satellite‐based techniques are used to chart AIS imbalance, measurements of surface elevation change (e.g., Pritchard et al., 2009; Wingham et al., 1998), of ice flow (e.g., Joughin et al., 1999; Rignot et al., 2011; Scambos et al., 1992), and of changing gravitational attraction (e.g., Luthcke et al., 2013; Velicogna & Wahr, 2006). Although these methods have different strengths and weaknesses, their estimates of ice sheet mass balance agree when common geographical regions, time intervals, and models of surface mass balance and glacial isostatic adjustment are used (Shepherd et al., 2018). When high‐resolution satellite measurements are combined with regional climate models (e.g., Bromwich et al., 2011; Melchior Van Wessem et al., 2018) or information on ice flow, it is possible to identify signals of ice sheet imbalance that are meteorological (Boening et al., 2012; Davis et al., 2005) and ice dynamical (Joughin et al., 2002; Rignot et al., 2005; Scambos et al., 2004; Shepherd et al., 2001; Sutterley et al., 2014) in origin.

Since 1992, the ERS‐1 (Wingham et al., 1998), ERS‐2 (Davis & Ferguson, 2004; Zwally et al., 2005), ENVISAT (Flament & Remy, 2012; Wingham et al., 2006; Zwally et al., 2015), and CryoSat‐2 (Helm et al., 2014; McMillan et al., 2014) satellite radar altimeters have measured changes in the AIS surface elevation at approximately monthly intervals. Between 2003 and 2009, the ICESat satellite laser altimeter also measured elevation changes during 18 campaigns (Pritchard et al., 2009; Zwally et al., 2015). The radar altimeter data constitute the longest continuous record of ice sheet wide change recorded by similar sensors with similar spatial and temporal sampling and are a unique resource for studying ice sheet imbalance. In this paper we combine their measurements to determine changes in the elevation and volume of the East Antarctic (EAIS) and West Antarctic (WAIS) ice sheets and parts of the Antarctic Peninsula ice sheet (APIS), over a 25‐year period. We examine local and regional trends within the principal drainage basins of each ice sheet, and we provide an estimate of change within areas that are only partially surveyed. Using a firn densification model (Ligtenberg et al., 2011) driven by a regional climate model (Melchior Van Wessem et al., 2018), we then adjust the measured elevation changes to account for signals associated with fluctuations in snowfall, which allows us to locate and chart the evolution of ice sheet dynamical imbalance.

## 2 Ice Sheet Elevation Change

We developed time series of surface elevation change across the AIS and within its principal drainage units from ERS‐1, ERS‐2, ENVISAT, and CryoSat‐2 radar altimeter observations recorded between May 1992 and May 2017. Over 800 million altimeter measurements were included in our analysis, sampling 86%, 76%, and 79% of the APIS, EAIS, and WAIS, respectively. To compute elevation, the altimeter range measurements were corrected for the lag of the leading‐edge tracker, dry atmospheric mass, water vapor, the ionosphere, ocean loading tide, and temporal variations in surface scattering. We also adjusted for elevation changes associated with glacial isostatic adjustment using the IJ05_R2 model (Ivins et al., 2013). The surface elevation change time series were computed (McMillan et al., 2014; Zwally et al., 1989) within regularly spaced grid cells and during fixed time intervals (epochs). In the first instance, we generated time series from each satellite mission independently, and we then combined them by adjusting for the bias occurring during periods of mission overlap.

Although the majority of the grounded ice sheet was surveyed, a proportion fell beyond the southern limit of the satellite orbits (1% for CryoSat‐2 and 21% for other missions). In other areas, where the altimeters failed to track rugged terrain (<2%) and in gaps between the satellite ground tracks, we interpolated the observed elevation changes using a triangulation scheme (Shepherd et al., 2001) at each epoch. We then computed elevation trends over discrete time intervals in each grid cell, within ice sheet drainage basins (Zwally et al., 2012) and also within the limits of the EAIS and the WAIS. We did not compute a regional assessment for the APIS because its northern drainage basins were too sparsely sampled. Finally, we modeled rates of elevation change in the remaining (mainly coastal) regions using local empirically determined relationships with rates of ice flow.

To estimate the uncertainty of our elevation‐change time series, we summed systematic and time‐varying errors at each epoch, plus errors arising from the bias adjustment we apply to align each satellite mission. We estimated systematic uncertainties as the standard error of the long‐term rate of elevation change in each region. Time‐varying uncertainties at each epoch were computed as the average standard error of elevation measurements within the aggregated pixels. Uncertainties associated with the inter‐mission bias were estimated as the standard deviation of differences between modeled elevations during a 2‐year period centered on each mission overlap. At each epoch, uncertainties from all three error sources were summed in quadrature to estimate the overall error.

Alternative approaches have been proposed for certain stages in the processing of ice sheet elevation change. Key examples include (i) the method of determining the satellite range, which has been done with a variety of waveform retracking routines (e.g., Davis, 1997; Helm et al., 2014; Nilsson et al., 2016) and with a variety of adjustments to account for temporally correlated fluctuations in the radar echo shape (e.g., Davis & Ferguson, 2004; Flament & Remy, 2012; Wingham et al., 1998; Zwally et al., 2005) and (ii) the approach to forming elevation change time series, which has been done at orbit crossing points (Wingham et al., 1998; Zwally et al., 1989), along repeated ground tracks (Flament & Remy, 2012; Pritchard et al., 2009; Sorensen et al., 2011), and within fixed geographical regions (Helm et al., 2014; McMillan et al., 2014). While no approach has led to substantially different patterns of ice sheet elevation change in Antarctica, we nevertheless investigated the impact of many by testing alternative scenarios that employ different approaches to (i) form the initial time series, (ii) correct for correlated trends in echo shape, and (iii) account for spatial and temporal omission.

**Figure 1:**
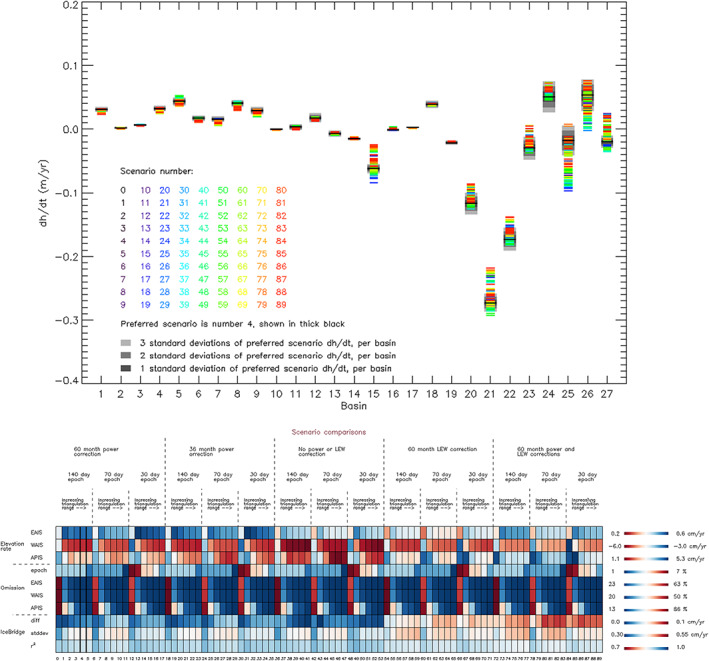
(top) Average rate of elevation change in AIS drainage basins (Zwally et al., 2012) between 1992 and 2017 derived from 90 alternative processing scenarios. The optimal scenario is represented by a thick black line, with its estimated 1*σ*, 2*σ*, and 3*σ* uncertainty range shaded in dark, mid, and light gray, respectively. In most basins, the average elevation rate and the spread among scenarios are close to zero. Basins showing the greatest spread among scenarios are either sparsely sampled (15 and 24 to 27) or include changes in ice thickness that are large by comparison to the spread (20 to 22). (bottom) Evaluation of the alternative processing scenarios: (i) in producing average ice sheet elevation rates (top three rows, see also Figure S9), (ii) in relation to their temporal and spatial sampling (middle four rows, see also Figure S3), and (iii) in relation to their difference to precise airborne laser altimetry (Studinger, 2014; bottom three rows, see also Figure S11). Against these metrics, scenario 4 (thick border) is identified to be the optimal elevation change solution. AIS = Antarctic Ice Sheet; EAIS = East AIS; WAIS = West AIS; APIS = Antarctic Peninsula ice sheet; LEW = leading edge width.

To evaluate the processing scenarios (Figure 1), we considered their ability to match independent estimates of elevation change derived from precise airborne laser altimetry and to address undersampling of signal at northerly latitudes where the satellite orbit ground tracks diverge. Against these benchmarks, the optimal scenario among those we have considered uses a plane fit elevation change solver, averages measurements in 140‐day epochs, interpolates elevation changes over distances of 20 km to fill remaining gaps, uses a 60‐month period as the basis of corrections to compensate for correlated fluctuations in elevation and backscattered power, and does not apply a leading edge width correction. When compared to 544,422 repeat airborne laser altimetry measurements (Studinger, 2014), the optimal elevation change solution differed by 0.7 cm/year, on average, with CryoSat‐2's interferometric mode showing 15% lower variance thanks to its smaller ground footprint and ability to more precisely locate the angle to the surface reflection. The average difference is smaller than the estimated certainty of our 25‐year rate of elevation change for WAIS (1.0 cm/year)—an area of comparable size—which suggests that our error model adequately captures the principal sources of uncertainty. Moreover, of the 90 alternative scenarios we considered, 86% and 83% produced EAIS and WAIS elevation trends that fell within the estimated 1‐sigma (67%) uncertainty of our optimal solution, respectively, which suggests that our error model also adequately captures variance associated with the processing methods we have tested. For more details on the altimetry methods, see Supporting Information S1.

**Figure 2:**
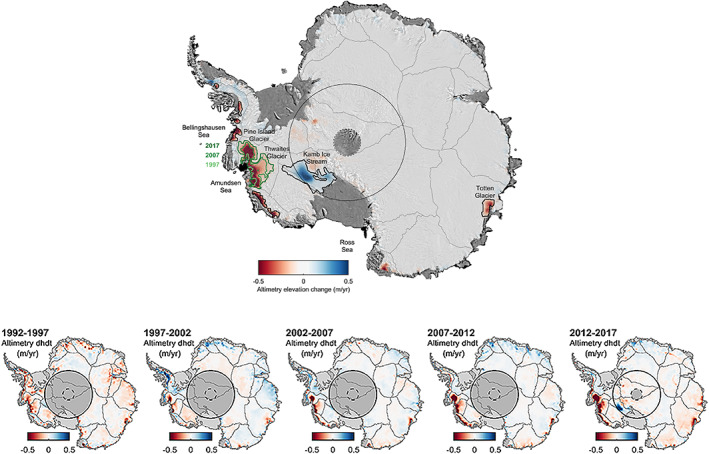
Average rate of Antarctic Ice Sheet elevation change between 1992 and 2017 (top) and within successive 5‐year intervals (bottom) from satellite radar altimetry, smoothed with a 100 km Gaussian filter. Black circles at the pole indicate the southern limit of the CryoSat‐2 (dashed) and other (solid) satellite orbits. Gray boundaries show glacier drainage basins (Zwally et al., 2012). Boundaries show areas of dynamical imbalance that do not (black) and do (green) evolve over time.

Although most of the AIS surface has changed little in elevation over the past 25 years (Figure 2), there are clear patterns of thinning and thickening in coastal sectors—especially in WAIS and the APIS. Some of these signals have been identified in shorter records (Flament & Remy, 2012; McMillan et al., 2014; Pritchard et al., 2009; Shepherd et al., 2002; Shepherd & Wingham, 2007; Wingham et al., 1998; Zwally et al., 2005) and are now better defined because our time series is long in comparison to the period of snowfall fluctuations, which are typically decadal or less in Antarctica (Shepherd et al., 2012; Wouters et al., 2013). Most of the significant changes are coincident with glaciers and ice streams (Rignot et al., 2011). In some cases, the origins of these signals have been related to changes in ice flow through independent observations, for example, slowdown of glaciers at the Siple Coast (Anandakrishnan & Alley, 1997; Scheuchl et al., 2012) is driving ice sheet thickening in this sector, and speedup of glaciers draining into the Amundsen Sea (Joughin et al., 2003; Mouginot et al., 2014) and of Fleming Glacier (Rignot et al., 2005) is causing thinning. In other cases, ice sheet thinning has been related to dynamical imbalance through indirect measures such as excessive ice discharge or grounding line retreat; Totten Glacier (Li et al., 2015) and several Bellingshausen Sea glaciers (Christie et al., 2016) fall into this category, as a contemporary speedup has not been recorded.

Elsewhere, there are signals of ice sheet elevation change that are distal to areas of fast ice flow. In Dronning Maud Land (basins 5 to 8), a broad pattern of modest ice sheet thickening spans much of the coastline and stretches several hundred kilometers inland; this has been associated with sharp increases in snowfall that occurred between 2009 and 2012 (Boening et al., 2012). Modest thickening of the ice sheet to the west of Ronne Ice Shelf (basin 1) may also be meteorological in origin. The magnitudes of these signals are small (5 to 10 cm/year) and do not coincide with areas of rapid ice flow or of known ice dynamical imbalance. However, the same cannot be said for stronger patterns of ice sheet thickening in the upper reaches of drainage catchments of the Bellingshausen Sea sector (basins 23 and 24) or of ice sheet thinning inland of the southern Siple Coast ice streams (basins 17 and 18); these changes are adjacent to areas where ice flow units are changing in thickness and complicate their interpretation. In the southwestern Antarctic Peninsula, for example, a modest speedup of glaciers flowing into George VI Ice Shelf accounts for only a fraction of the observed coastal deflation (Hogg et al., 2017) and, similarly, thinning of glaciers flowing into the Getz Ice Shelf is greater than estimated increases in ice discharge alone (Chuter et al., 2017). In these two sectors, the pattern of elevation change reflects a complex mix of surface processes and ice dynamical imbalance.

## 3 Ice Dynamical Imbalance

Changes in AIS elevation arise predominantly due to fluctuations in accumulation and ice flow, which occur at the densities of snow and ice, respectively (Wingham, 2000). These processes can be distinguishable in altimeter records due to their coincidence with areas of rapid or changing ice flow or due to their persistence over periods that are long in comparison to expected snowfall fluctuations. To discriminate them, we adjusted the satellite elevation changes to account for fluctuations in surface mass balance by removing model estimates of the firn layer thickness change (Ligtenberg et al., 2011; Melchior Van Wessem et al., 2018). We then classified regions exhibiting correlated patterns of sustained and significant thickening or thinning relative to the firn thickness changes as areas of ice dynamical imbalance. In some places, ice dynamical imbalance has spread inland (e.g., Konrad et al., 2017), and so we allowed these areas to grow over time if a significant accelerated elevation trend is present. Using this approach, there is, for example, a ~200,000‐km^2^ increase in the area of dynamical imbalance in the Amundsen Sea sector over the 25‐year survey period, which corresponds to a 150‐ to 300‐m/year decrease in the average speed (Rignot et al., 2011) of ice that is thinning. Altogether, 56,950, 415,175, and 17,900 km^2^ of the EAIS, WAIS, and APIS were identified in this scheme to be in a state of dynamical imbalance by the end of 2017. Elsewhere, we assumed that elevation changes were caused by fluctuations in surface mass balance, though potential signals of dynamical imbalance that are modest, highly localized, abrupt, or episodic may not be fully resolved. For more details on the ice dynamical imbalance methods, see Supporting Information S1.

Our classification scheme identifies long‐term ice dynamical imbalance in 10 drainage basins, including two where the area of imbalance grows over time. At the Siple Coast, ice thickening is steady and uniform across three southern ice flow units which have inflated by 232 ± 27 cm, on average, over the 25‐year survey. In contrast, thinning of the Pine Island, Thwaites, and Totten glaciers increases toward the ice sheet margin, peaking at 80 ± 2, 122 ± 1, and 26 ± 1 m, respectively, at their termini. These glaciers drain the principal marine‐based and ocean‐terminating sectors of Antarctica, a geometrical configuration that is theoretically unstable (Schoof, 2007) and is in numerical simulations highly sensitive to ice marginal perturbations (Joughin et al., 2014; Payne et al., 2004). There is also evidence of ocean‐driven melting at their termini (Dutrieux et al., 2014; Jacobs et al., 2011; Rintoul et al., 2016). On average, the sections of the Pine Island, Thwaites, and Totten glaciers identified to be in a state of dynamical imbalance thinned at rates of 45 ± 5, 49 ± 5, and 25 ± 3 cm/year, respectively, between 1992 and 2017, whereas the remainder of their catchments were relatively stable. At the Totten glacier, dynamical imbalance affects just 3.6% of the drainage basin, and we found little evidence that either this or the rate of elevation change have changed over the 25‐year survey. In contrast, ice drawdown within the Pine Island and Thwaites glacier drainage basins has spread rapidly inland and now affects the majority (51% and 68%, respectively) of their catchments.

## 4 Ice Sheet Mass Balance

We used our classification of ice dynamical imbalance as the basis of a spatially resolved mass balance calculation, as it allows us to separate elevation changes predominantly occurring at the densities of snow and ice. Our classification is an improvement over previous schemes because it uses model estimates of firn thickness change to locate the change in ice thickness, rather than attributing elevation changes within entire drainage sectors (Wingham et al., 1998) or otherwise defined regions (Shepherd et al., 2012) to ice. It does not, however, account for potentially coincident signals of meteorological and dynamical imbalance, and this is an acknowledged shortcoming. Nevertheless, although making an explicit, model‐based correction for snowfall fluctuations (Zwally et al., 2015) is in principal ideal, our classification scheme remains preferable because (i) firn models are not well matched with satellite radar altimeter elevation changes, because (ii) their application leads to spurious signals of ice dynamical imbalance in areas changing at rates below the certainty of the altimeter and firn model trends, and because (iii) the standard deviation between the resulting mass trends is 43% lower when compared to independent estimates derived from satellite gravimetry (Table S4). We instead treated snowfall as an additional source of elevation change uncertainty in mass balance calculations by summing an estimate of its variability (Table 1) in quadrature with the satellite elevation trend uncertainties. For more detailed on the mass balance methods and evaluation, see Supporting Information S1.

**Table 1:** The Observed Area, Mean Accumulation Rate, Estimated Snowfall Variability, Average Elevation Rate, and Average Mass Balance of AIS Drainage Basins Between May 1992 and May 2017

Drainage basin	Area (km^2^)	Mean ice accumulation rate (cm/year)	Snowfall variability (cm/year)	Elevation rate (cm/year)	Average mass balance (Gt/year)
1	465,525	29	4.2	3.1 ± 0.3	5.7 ± 2.1
2	741,575	7	1.6	0.3 ± 2.3	0.7 ± 6.2
3	1,501,700	5	0.4	0.7 ± 0.1	3.6 ± 0.6
4	239,475	22	3.2	3.3 ± 0.4	3.1 ± 0.6
5	184,750	21	4.3	4.4 ± 0.6	3.2 ± 0.6
6	599,025	13	1.4	1.8 ± 0.3	4.1 ± 0.8
7	493,875	18	2.3	1.6 ± 0.3	3.3 ± 0.9
8	162,250	23	4.7	4.1 ± 0.4	2.7 ± 0.5
9	145,725	15	3.1	3.0 ± 0.7	1.7 ± 0.5
10	893,375	5	0.4	0.0 ± 0.2	0.0 ± 0.8
11	252,625	7	1	0.4 ± 0.4	0.3 ± 0.4
12	722,525	23	2.1	1.8 ± 0.4	5.6 ± 1.3
13	1,108,875	23	2.1	−0.7 ± 0.4	−8.2 ± 2.0
*14*	711,675	21	2.1	−1.5 ± 0.2	−4.7 ± 1.0
15	123,675	25	5.3	−6.2 ± 1.5	−2.9 ± 0.8
16	258,825	5	0.8	−0.1 ± 0.3	−0.1 ± 0.3
17	1,769,850	5	0.3	0.3 ± 0.1	2.9 ± 0.8
***18***	253,975	13	3.4	3.9 ± 0.7	9.3 ± 1.5
***19***	358,525	14	1.6	−2.1 ± 0.3	−1.6 ± 0.8
*20*	177,625	79	12.9	−11.6 ± 1.0	−16.4 ± 4.0
***21***	210,600	56	8.5	−27.2 ± 0.7	−46.1 ± 7.2
***22***	208,025	43	6.4	−17.2 ± 0.8	−28.4 ± 6.2
*23*	73,925	99	18.9	−2.9 ± 1.3	−4.3 ± 2.1
*24*	99,525	92	17.1	5.1 ± 1.8	0.2 ± 1.5
25	35,425	202	40.5	−1.7 ± 2.9	−1.5 ± 1.3
*26*	41,500	109	23.4	5.4 ± 5.2	
27	51,275	44	7.9	−2.0 ± 2.3	
EAIS	9,909,800	12	0.5	0.6 ± 0.1	16.3 ± 5.5
WAIS	1,748,200	37	2.8	−5.6 ± 0.3	−81.9 ± 12.1
AP	227,725	101	16.6	2.7 ± 2.0	

*Note*. Basins with regions of identified ice dynamical imbalance are italicized, and where elevation changes also exceed expected snowfall variability are in bold. AIS = Antarctic Ice Sheet; EAIS = East AIS; WAIS = West AIS; AAntarctic Peninsula.

Seven drainage basins are situated partly within the region that falls beyond the satellites' southern orbital limits. Although this area represents a sizeable fraction (21%) of the continental ice sheet for ERS‐1, ERS‐2, and ENVISAT, the broader pattern of elevation change within the Antarctic interior has remained stable over the survey period and is consistent with the changes recorded farther south by CryoSat‐2 since 2010; that is, the Kamb, Whillans, and Mercer ice streams have thickened, steadily, while little change has occurred elsewhere. Our estimate of the ice sheet interior mass balance is based on elevation trends recorded in the southernmost degree of latitude sampled and puts the region in a state close to balance—gaining 1.7 ± 0.1 Gt/year, on average, since 1992. At the continental scale, we estimate that the EAIS and WAIS changed in mass at average rates of +16 ± 6 and −82 ± 12 Gt/year, respectively, between 1992 and 2017 (Table 1). These values are in close agreement with a recent community assessment of 24 satellite altimetry, gravimetry, and mass budget surveys (Shepherd et al., 2018), which put the EAIS and WAIS mass trends at +5 ± 46 and −94 ± 27 Gt/year over the same period. Our estimates of ice sheet mass balance are also in close agreement with estimates (Groh & Horwath, 2016) derived from GRACE satellite gravimetry; across the 23 drainage basins included in our survey and over a common 13‐year period (2002 to 2016), the root mean square difference between the two estimates is 4.9 Gt/year.

**Figure 3:**
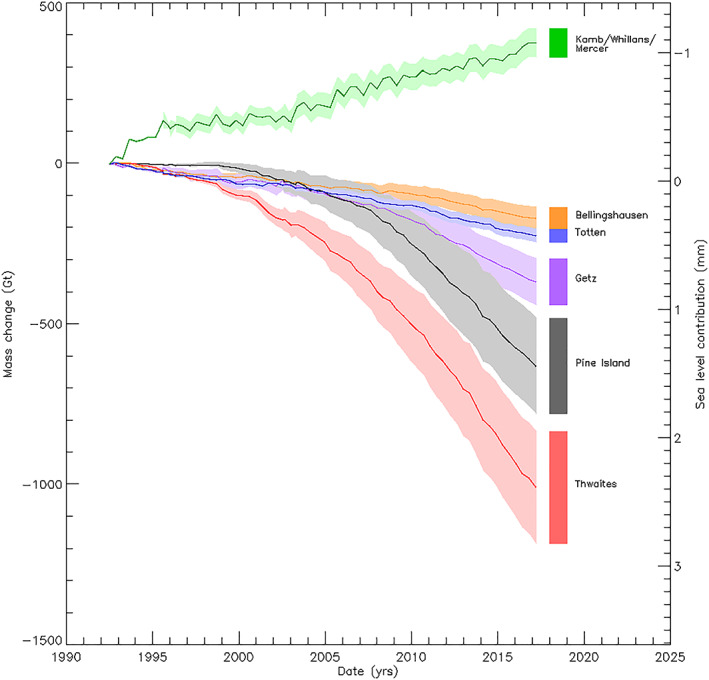
Mass change and sea level contribution of areas in a state of ice dynamical imbalance (see Figure 2 for locations) and their estimated 1*σ* uncertainty (shaded area).

By far, the largest signal of imbalance has occurred in the Pine Island and Thwaites glacier drainage basins (Figure 3), which have lost mass at average rates of −28 ± 6 and −46 ± 7 Gt/year, respectively, since 1992 (Table 1). Despite a steady gain of 13 ± 4 Gt/year within the southern Siple Coast ice streams (Table S3), Amundsen Sea sector glaciers dominate the overall mass balance of WAIS which has lost an estimated 1,851 ± 133 Gt of ice over the 25‐year survey. Although we have identified an area of persistent thinning at Totten glacier as being dynamical in origin, these losses (9 ± 1 Gt/year) are small by comparison to changes in WAIS. They are also offset by modest ice sheet thickening across the remainder of the EAIS which, overall, has gained an estimated 407 ± 161 Gt of snow and ice since 1992.

Accelerating mass loss from the Amundsen Sea Sector has been documented in a wide range of satellite altimetry (Thomas et al., 2004; Wingham et al., 2009), mass budget (Medley et al., 2014; Rignot et al., 2008), and gravimetry (Bouman et al., 2014; Velicogna et al., 2014) surveys, and in comparison, the techniques have shown close agreement (Shepherd et al., 2012; Sutterley et al., 2014). We examined this signal by calculating mass trends within the regions of ice dynamical imbalance of the Pine Island and Thwaites glacier drainage basins over 5‐year intervals (see Table S3). For comparison, we repeated this calculation also for the Totten and Siple Coast glaciers, where the area of dynamical imbalance has remained static. Rates of ice mass loss from the Pine Island and Thwaites glaciers have increased progressively over time, rising from 2 ± 1 and 12 ± 1 Gt/year, respectively, between 1992 and 1997, to 55 ± 4 and 76 ± 6 Gt/year, respectively, during the latter period of our survey (2012 to 2016). A consequence of this inland spreading is that over half (59%) of the Amundsen Sea sector is now in a state of dynamical imbalance. In keeping with the findings of previous studies (Medley et al., 2014; Rignot et al., 2008; Rignot et al., 2014), our data confirm that the Pine Island and Thwaites Glacier are by far the largest regional contributors to global sea level rise. In contrast, rates of mass loss and gain from the Totten and Siple Coast glaciers have remained relatively stable over time.

## 5 Conclusions

Our ERS‐1, ERS‐2, ENVISAT, and CryoSat‐2 radar altimetry time series provides a comprehensive assessment of the spatial and temporal pattern of AIS elevation change between 1992 and 2017. When combined with model estimates of firn thickness change (Ligtenberg et al., 2011; Melchior Van Wessem et al., 2018), the 25‐year altimeter record enables long‐term trends in ice thickness to be separated from short‐term snowfall variability. While the majority of the ice sheet surface has remained stable over the survey period, areas of ice dynamical imbalance are now clearly apparent across many sectors of the continent. We estimate that 0.6%, 23.7%, and 7.9% of the EAIS, WAIS, and APIS, respectively, are now in a state of dynamical imbalance. Assuming changes in these areas have occurred at the density of ice and the remainder at the density of snow, we estimate that, together, the EAIS and WAIS changed in mass at an average rate of −66 ± 18 Gt/year between 1992 and 2017. This equates to an average 4.6 ± 1.2 mm contribution to global sea level over the same period, a value that is comparable to estimates determined using other approaches (Shepherd et al., 2018). Ice losses from the Amundsen Sea sector of WAIS have increased progressively over the course of our survey and were 5 times greater in the final decade of our survey than during the initial decade. A future goal is to develop an optimal altimetry ice sheet mass balance solution that fully incorporates model estimates of firn thickness and surface mass balance.

## Supporting information

Supporting Information S1
