# Supplementary material for: Trends in Antarctic Ice Sheet Elevation and Mass
Source: Geophys Res Lett. 2019 Jul 24;46(14):8174–83. doi: 10.1029/2019GL082182 (PMC9285922; doi:10.1029/2019GL082182)
Supplement: Supplementary file 1 — Supporting Information S1 [file GRL-46--s001.docx]

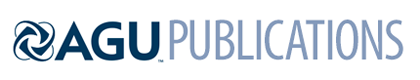


*Geophysical Research Letters*

Supporting Information for

**Separating trends in Antarctic ice sheet elevation and mass**

Andrew Shepherd^1*^, Lin Gilbert^2^, Alan S. Muir^2,3^, Hannes Konrad^1^, Malcolm McMillan^1^, Thomas Slater^1^, Kate H. Briggs^1^, Aud V. Sundal^1^, Anna E. Hogg^1^, Marcus Engdahl^4^

1. Centre for Polar Observation and Modelling, School of Earth and Environment, University of Leeds, LS2 9JT, UK

2. Mullard Space Science Laboratory, Department of Space & Climate Physics, University College London, WC1E 6BT, UK

3. Centre for Polar Observation and Modelling, Department of Earth Sciences, University College London, WC1E 6BT, UK

4. ESA-ESRIN, Via Galileo Galilei, Frascati (Roma), I-00044, Italy

**Contents of this file**

Text S1 to S9

Figures S1 to S13

Tables S1 to S3

**Introduction**

This document provides supporting information to describe the methods we use for (S1) computing single-mission ice sheet elevation changes, for (S2) computing multi-mission ice sheet elevation changes, for (S3) the treatment of unobserved areas, for (S4) generating alternative elevation change scenarios, for (S5) estimating snowfall variability, for (S6) estimating firn layer thickness change, for (S7) locating areas of ice dynamical imbalance, for (S8) computing ice sheet mass trends, and for (S9) evaluating the ice sheet elevation and mass trends.

**Text S1. Computing Single-Mission Ice Sheet Elevation Changes**

The raw elevation data were from radar altimeters mounted on four satellites, ERS-1, ERS-2, ENVISAT and CryoSat-2. These satellites have provided continuous coverage of the Antarctic ice sheets since May 1992. ERS-1 and ERS-2 data were surfaces flagged as continental ice, when the satellite was in ice tracking mode, from ‘Reprocessing of altimeter products for ERS’ (REAPER) level 2 data files. ENVISAT data were surfaces flagged as continental ice, when the satellite was in 320 Mhz tracking mode, from level 2 radar altimeter geophysical data record v2.1 data files. CryoSat-2 data were surfaces flagged as land/ice from baseline C level 2 low rate mode and synthetic aperture radar interferometry mode data files. For all ERS-1, ERS-2, Envisat, and CryoSat-2 low resolution model (LRM) data, the altimeter waveforms were processed using a Threshold offset Centre Of Gravity (TCOG) retracker. For CryoSat-2 synthetic aperture radar interferometry mode, the altimeter waveforms were retracked using the model described in Wingham *et al.* (2006c). In each case, the measurements used were time, slope-corrected geographic location, slope- and geophysically-corrected height, backscatter power and orbit heading (ascending or descending). The geophysical corrections used were the dry tropospheric correction, the wet tropospheric correction, the ionospheric correction, the solid Earth tide and the ocean loading tide. ERS-1, ERS-2 and CryoSat-2 data files included the geophysical corrections in their height measurements, while for ENVISAT they were supplied separately and applied during data ingestion. Due to an error in some ENVISAT data files, a better dry tropospheric correction was obtained from an auxiliary set of point target response files. We used an external model (Iijima et al., 1999) to adjust Envisat data for propagation of the radar signal through the ionosphere after the secondary S-band altimeter failed in 2008. A correction was also applied to the elevation measurements to account for the effects of post-glacial rebound, using the IJ05_R2 model (Ivins et al., 2013).

Elevation changes were computed for each mission, separately, on a 5 km square grid of the Antarctic region, using a polar stereographic projection based on the World Geodetic System 1984 ellipsoid, with true scale latitude 71°S and central longitude 0°E. The grid contains 1128 columns and 968 rows, with bottom left corner at x = -2.82e6 m and y = -2.42e6 m. Each cell in the grid was treated separately. Data falling within grid cells were only used to compute elevation changes if they contained 15 or more individual measurements. First a surface model was fitted to the cell data, using a Levenberg-Marquardt least squares fitting method. The model equation is

|  | $z\left( x,y,h \right)=z_{m}+a_{0}x+a_{1}y+a_{2}x^{2}+a_{3}y^{2}+a_{4}xy+a_{5}h+a_{6}t$ | Eq. (S1) |
| --- | --- | --- |

where $z$ is height, $x$ is the polar stereographic easting coordinate, $y$ is the polar stereographic northing coordinate, $h$ is the satellite heading (set as binary), and $t$ is the time of the elevation measurement in years. The coefficients of the fit were solved using the least squares fit to measurements falling within each grid cell. Measured heights more than two standard deviations from the modelled height were discarded, and this procedure was repeated until either no outliers or fewer than fifteen data points remained (in which case the results in the grid cell were not used).

A second model was then fitted to the slope- and satellite heading-corrected elevation anomalies emerging from each mission plane fit solution to remove residual, short-period fluctuations correlated with changes in backscattered power that arise in radar altimeter measurements over continental ice sheets (Wingham et al., 1998). This model was applied in a separate step to ensure that it did not interfere with the spatial and temporal elevation fit. It was again determined using a Levenberg-Marquardt least squares, with an equation of the form

|  | $p=a_{0}+a_{1}t+a_{2}h$ | Eq. (S2) |
| --- | --- | --- |

where $p$ is the backscatter power, $t$ is the time of the measurement in years, and $h$ is the satellite heading. A time series of backscatter power was reconstructed using this model fit and the anomalies, and 5-year trends in ${dp}/{dz}$ were computed centred on the mid-point of each mission by matching the power and elevation anomaly time-series. These periods were chosen due to their relative stability in terms of orbit manoeuvres, outages and on-board changes. The fitting procedure was again iterated to remove outliers more than two standard deviations from the modelled value, either until there were none or more than three iterations had occurred (in which case the results were not used).

Our approach allowed time series of elevation change to be formed within 25 km^2^ or coarser regions, and we aggregated the measurements into 72 separate 140-day epochs across the four satellite missions. In each grid cell, the average residual height within each epoch was calculated using a resistant mean by discarding data more than two standard deviations from the median and compensating for the truncation with an approximation formula. During each epoch, empty grid cells were filled wherever possible by triangulation, up to a limiting side-length for a given triangle of 100 km.

For any given satellite mission, we estimated the uncertainty at each epoch of an elevation change (*dz*) time series as a combination of systematic and time-varying sources of error. Systematic errors were defined as those that may impact the long-term trend in elevation, and were estimated from the standard error of the rate of surface elevation change (*dz/dt*) that was derived from each respective time series. Sources of systematic error may include spatially coherent changes in elevation that are not represented by the functional form of our model, such as short-lived accumulation events or changes driven by snowpack characteristics that are not accounted for by our empirical backscatter model (Equation S2). For each time series, we cumulatively summed the systematic uncertainty at each epoch, so that the contribution from this component grows linearly with time. Additional, time varying uncertainty may arise due to errors that affect individual epochs, and impinge on our ability to determine the regionally averaged elevation anomaly at that particular time. This term is influenced by factors such as measurement precision and non-uniform spatial sampling, and we quantified its influence based upon the dispersion of contributing measurements at each individual epoch. Specifically, for every epoch within any given time series, we computed the regional average of the standard error of *dz* measurements within all contributing pixels. In contrast to the systematic term, we assume the time varying component to be temporally uncorrelated, and so at any given epoch we added all preceding epoch uncertainties in quadrature. Finally, we summed the systematic and time-varying contributions in quadrature, to determine an estimate of the overall elevation change uncertainty at each epoch.

**Text S2. Computing Multi-Mission Ice Sheet Elevation Changes**

To produce continuous, multi-mission time-series of height change, biases have to be accounted for between missions. In all cases, the objective was to align ERS-2, ENVISAT, and CryoSat-2 time-series with ERS-1. First, a model was defined for the shape of each time-series taking the form of a seasonal cycle imposed on a linear gradient. The model equation is

|  | $z=a_{0}+a_{1}t+a_{2}\sin\left( 2\pi t+a_{3} \right)$ | Eq. (S3) |
| --- | --- | --- |

where $z$ is the height change and $t$ is the average time at each epoch in the series, in years. For each mission, the model coefficients were solved for using a Levenberg-Marquardt least-squares fit applied to sections of data centered on the mid-times between mission end and start dates. The lengths of each section varied due to the duration of the mission overlap, and ranged from 1 to 3.5 years. For each overlapping pair of missions, the bias was calculated as the median value of the difference between modelled height anomalies over a common, 2-year period, centred on the mid-point of the mission end and start dates (e.g. Figure S1).

| 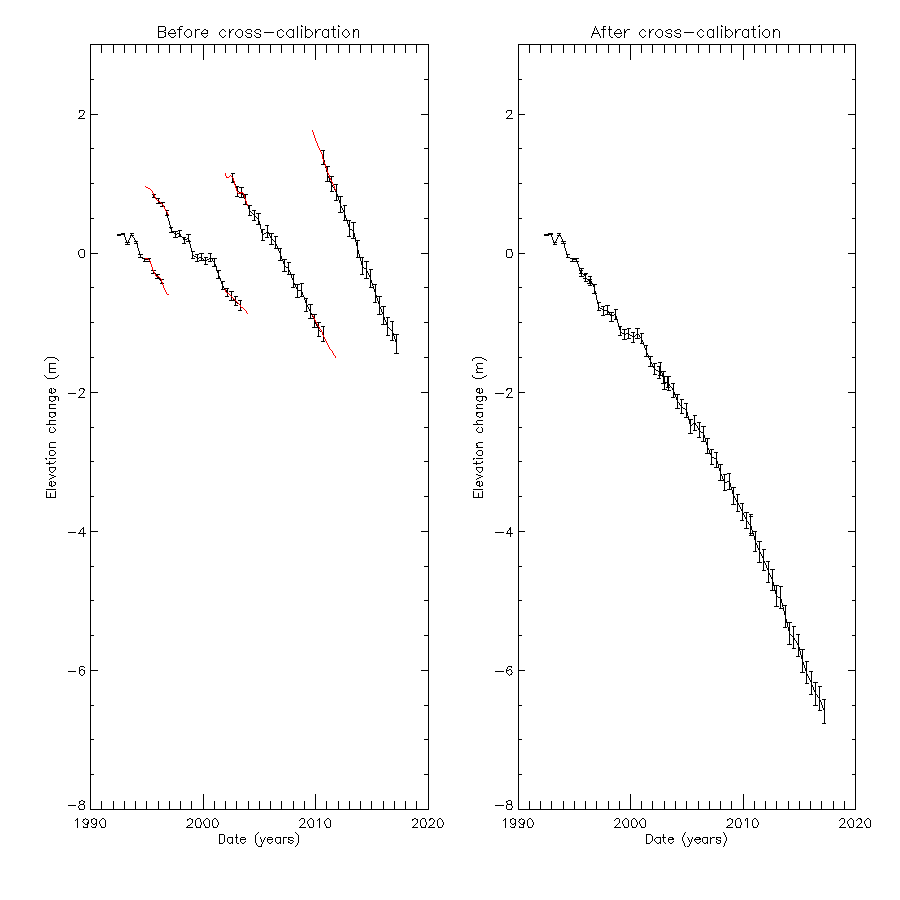 |
| --- |
| **Figure S1.** Example elevation trends computed from single mission time series (left) and the multi-mission ensemble (right) computed after adjusting for the bias arising at mission overlap periods (shown in red). |

The biasing method was applied to elevation changes within individual grid cells (pixel cross-calibration), and to averages computed over larger regions of interest (termed basin cross-calibration), including areas of ice dynamical imbalance, drainage basins (e.g. Figure S2), and ice sheets. The certainties of the bias corrections improve as the area of interest increases due to the volumes of data included in the model fits. Nevertheless, when aggregated over drainage basins, the multi-mission elevation changes computed over grid cells differ from those computed over larger regions by less than 1 mm/yr, on average. Any residual errors resulting from our method of accounting for inter-satellite biases will introduce additional uncertainty into the multi-mission time series. To estimate the associated uncertainty, we computed the standard deviation of the differences between the modelled elevations from the two satellite missions. This essentially measures the precision with which we can align the two missions, based upon the variance of the respective modelled elevations within the defined overlap period. The biasing uncertainty was set to zero for the first mission in our time series (ERS-1), as by definition no multi-mission adjustment is required, and then increases at each subsequent inter-mission boundary. Specifically, at each epoch the biasing uncertainties arising from all preceding inter-mission overlap periods were summed in quadrature. The total multi-mission uncertainty at each epoch was then computed by summing the single mission uncertainty (described above) and the biasing uncertainty in quadrature. Finally, the uncertainty on the multi-mission rate of elevation change was computed by dividing the total uncertainty accumulated at the end of the time series by the duration of the record, to ensure that all components of the uncertainty budget are taken into account within the resulting trend estimate.

| 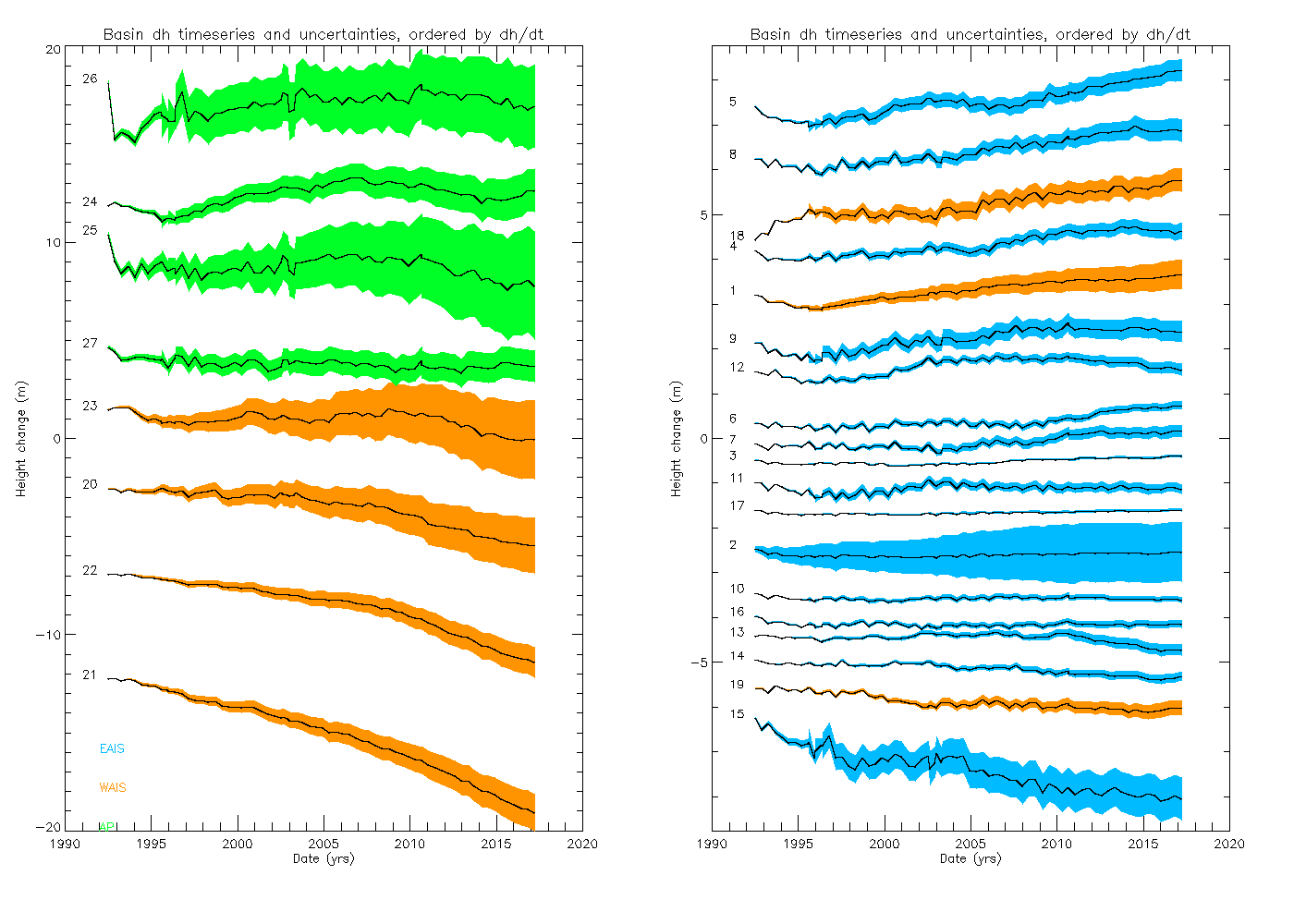 |
| --- |
| **Figure S2.** Relative elevation change of Antarctic ice sheet drainage basins (see Figure 2) and their estimated cumulative uncertainty (shaded area) as determined from ERS-1, ERS-2, ENVISAT, and CryoSat-2 satellite radar altimeter data. The drainage basins are colour coded according to the ice sheet in which they reside, and for compactness are arranged according to the magnitude of their average elevation trend with constant elevation offsets to ensure they are separate. Drainage basins showing significant variability or change are located in either West Antarctica or at the Antarctic Peninsula. |

**Text S3. Treatment of Unobserved Areas**

Although the elevation-change time-series survey the vast majority (98%) of the continental ice sheet area falling within the satellite orbital limits, some places are omitted where gaps arise between the satellite ground tracks, where the altimeters fail to track rugged terrain, and where the mission cross calibration failed, locally. We employed an interpolation and extrapolation scheme to provide continuous coverage of elevation trends in these areas which, in turn, allowed us to make basin-wide estimates. Of the 475,429 grid cells that comprise the grounded ice sheet, 374,115 were sampled by the 25-year satellite altimeter survey and a further 95,390 were sampled by CryoSat-2 south of the ERS/ENVISAT orbital limit. Elevation changes in 9,549 grid cells within 100 km of the ice sheet margin were estimated using an interpolation guided by ice velocity data, and in 239 other grid cells were estimated using the local drainage basin mean. For the purpose of mass balance calculation (see Text S8), elevation changes in 101,314 grid cells south of the ERS/ENVISAT orbital limit were additionally estimated using an extrapolation of ERS-1, ERS-2, and ENVISAT measurements and observations acquired by CryoSat-2, in 9,549 grid cells within 100 km of the ice sheet margin were estimated using an interpolation guided by ice velocity data, and in 239 other grid cells were estimated using the local drainage basin mean.

A small proportion of the ice sheet (2.1%) was unsurveyed due to gaps in the satellite orbits or failure of the altimeter tracking. Although this region is small, it does include some ice marginal areas due to the northwards broadening of ground track spacing, and in some sectors the pattern of thinning is correlated with ice flow. Because of this, in areas falling within 100 km of the ice sheet margin we modeled elevation rates using an interpolation algorithm guided by the magnitude of ice velocity based on the continuous pattern of ice flow derived from the Berkeley Ice Sheet Initiative for Climate Extremes model (Cornford et al., 2013). In each basin, the mean elevation rate was calculated within 15 equally spaced velocity bands up to 300 m/yr, and we modelled elevation trends based on a linear fit to these data. Gaps elsewhere were filled using the average elevation rate of the remainder of each drainage basin. These interpolation steps allow complete coverage, and do not measurably affect the average elevation trend of the continent.

By far the largest area of data omission was the region south of the satellite orbital limits, 88°S for CryoSat-2 and 81.5°S for the earlier missions. Although this unobserved area represents a sizeable fraction (23%) of the continental ice sheet for ERS-1, ERS-2, and ENVISAT, the vast majority of it (95%) was surveyed by CryoSat-2, and so we used CryoSat-2 measurement to inform our assessment of changes in this region. Considering all missions, the broader pattern of elevation change within the Antarctic interior has remained stable, and is consistent with the changes recorded farther south by CryoSat-2 alone; most of the interior has changed little, but flow units at the southern margin of the Siple Coast – the Kamb, Whillans, and Mercer ice streams - are in a state of positive dynamical imbalance. To estimate elevation changes in regions beyond the satellite orbital limits, we divided the unobserved region into the inland and downstream catchments of the Kamb, Whillans, and Mercer ice streams (which are thinning and thickening, respectively, due to the dynamical imbalance) and the remainder of the interior. We then computed the average elevation trend within the observed portions of these areas in the annulus running from 80⁰S to 81⁰S, and we extrapolated these rates to the unobserved portions of the respective regions.

**Text S4. Generating Alternative Elevation Change Scenarios**

To investigate the stability of our elevation change solution we evaluated 90 alternative processing scenarios, each employing different methods (see Table S1). The scenarios tested included various combinations of using the echo backscattered power (Davis and Ferguson, 2004; Wingham et al., 1998) and the echo leading edge width (Michel et al., 2014) to correct for changes in radar penetration into the snowpack, different temporal aggregation periods, and different interpolation schemes to address the latitudinally-varying sampling of the satellite orbits (Shepherd et al., 2002). We also tested, separately, elevation changes determined at crossing points of the satellite orbit ground tracks (Zwally et al., 1989), as a comparison to the repeat-track approach (McMillan et al., 2014) that we employ as a baseline scheme. This set of scenarios covers many, but not all of the alternative processing solutions that have been proposed in the literature. In particular, we did not assess the impact of corrections based on parameters not included in all satellite level-2 data products, for example bespoke range retrackers (e.g. (Helm et al., 2014; Nilsson et al., 2016)) or penetration corrections based on the echo trailing edge slope (e.g. (Flament and Remy, 2012)); these scenarios are beyond the scope of this study, which is designed to establish an optimal elevation change solution that is sufficiently accurate to draw firm conclusions on the evolution of Antarctic ice sheet elevation change.

|  | Backscatter correction | | |  |  |  |  | Backscatter correction | | |  |  |
| --- | --- | --- | --- | --- | --- | --- | --- | --- | --- | --- | --- | --- |
| # | Power | LEW | Period (months) | Epoch (days) | Radius (km) |  | # | Power | LEW | Period (months) | Epoch (days) | Radius (km) |
| 0 | Y | N | 60 | 140 | 0 |  | 45 | N | N |  | 70 | 15 |
| 1 | Y | N | 60 | 140 | 5 |  | 46 | N | N |  | 70 | 20 |
| 2 | Y | N | 60 | 140 | 10 |  | 47 | N | N |  | 70 | 30 |
| 3 | Y | N | 60 | 140 | 15 |  | 48 | N | N |  | 30 | 0 |
| 4 | Y | N | 60 | 140 | 20 |  | 49 | N | N |  | 30 | 5 |
| 5 | Y | N | 60 | 140 | 30 |  | 50 | N | N |  | 30 | 10 |
| 6 | Y | N | 60 | 70 | 0 |  | 51 | N | N |  | 30 | 15 |
| 7 | Y | N | 60 | 70 | 5 |  | 52 | N | N |  | 30 | 20 |
| 8 | Y | N | 60 | 70 | 10 |  | 53 | N | N |  | 30 | 30 |
| 9 | Y | N | 60 | 70 | 15 |  | 54 | N | Y | 60 | 140 | 0 |
| 10 | Y | N | 60 | 70 | 20 |  | 55 | N | Y | 60 | 140 | 5 |
| 11 | Y | N | 60 | 70 | 30 |  | 56 | N | Y | 60 | 140 | 10 |
| 12 | Y | N | 60 | 30 | 0 |  | 57 | N | Y | 60 | 140 | 15 |
| 13 | Y | N | 60 | 30 | 5 |  | 58 | N | Y | 60 | 140 | 20 |
| 14 | Y | N | 60 | 30 | 10 |  | 59 | N | Y | 60 | 140 | 30 |
| 15 | Y | N | 60 | 30 | 15 |  | 60 | N | Y | 60 | 70 | 0 |
| 16 | Y | N | 60 | 30 | 20 |  | 61 | N | Y | 60 | 70 | 5 |
| 17 | Y | N | 60 | 30 | 30 |  | 62 | N | Y | 60 | 70 | 10 |
| 18 | Y | N | 36 | 140 | 0 |  | 63 | N | Y | 60 | 70 | 15 |
| 19 | Y | N | 36 | 140 | 5 |  | 64 | N | Y | 60 | 70 | 20 |
| 20 | Y | N | 36 | 140 | 10 |  | 65 | N | Y | 60 | 70 | 30 |
| 21 | Y | N | 36 | 140 | 15 |  | 66 | N | Y | 60 | 30 | 0 |
| 22 | Y | N | 36 | 140 | 20 |  | 67 | N | Y | 60 | 30 | 5 |
| 23 | Y | N | 36 | 140 | 30 |  | 68 | N | Y | 60 | 30 | 10 |
| 24 | Y | N | 36 | 70 | 0 |  | 69 | N | Y | 60 | 30 | 15 |
| 25 | Y | N | 36 | 70 | 5 |  | 70 | N | Y | 60 | 30 | 20 |
| 26 | Y | N | 36 | 70 | 10 |  | 71 | N | Y | 60 | 30 | 30 |
| 27 | Y | N | 36 | 70 | 15 |  | 72 | Y | Y | 60 | 140 | 0 |
| 28 | Y | N | 36 | 70 | 20 |  | 73 | Y | Y | 60 | 140 | 5 |
| 29 | Y | N | 36 | 70 | 30 |  | 74 | Y | Y | 60 | 140 | 10 |
| 30 | Y | N | 36 | 30 | 0 |  | 75 | Y | Y | 60 | 140 | 15 |
| 31 | Y | N | 36 | 30 | 5 |  | 76 | Y | Y | 60 | 140 | 20 |
| 32 | Y | N | 36 | 30 | 10 |  | 77 | Y | Y | 60 | 140 | 30 |
| 33 | Y | N | 36 | 30 | 15 |  | 78 | Y | Y | 60 | 70 | 0 |
| 34 | Y | N | 36 | 30 | 20 |  | 79 | Y | Y | 60 | 70 | 5 |
| 35 | Y | N | 36 | 30 | 30 |  | 80 | Y | Y | 60 | 70 | 10 |
| 36 | N | N |  | 140 | 0 |  | 81 | Y | Y | 60 | 70 | 15 |
| 37 | N | N |  | 140 | 5 |  | 82 | Y | Y | 60 | 70 | 20 |
| 38 | N | N |  | 140 | 10 |  | 83 | Y | Y | 60 | 70 | 30 |
| 39 | N | N |  | 140 | 15 |  | 84 | Y | Y | 60 | 30 | 0 |
| 40 | N | N |  | 140 | 20 |  | 85 | Y | Y | 60 | 30 | 5 |
| 41 | N | N |  | 140 | 30 |  | 86 | Y | Y | 60 | 30 | 10 |
| 42 | N | N |  | 70 | 0 |  | 87 | Y | Y | 60 | 30 | 15 |
| 43 | N | N |  | 70 | 5 |  | 88 | Y | Y | 60 | 30 | 20 |
| 44 | N | N |  | 70 | 10 |  | 89 | Y | Y | 60 | 30 | 30 |
| **Table S1.** Characteristics of the alternative processing scenarios (#) used to derive estimates of Antarctic ice sheet elevation change, including whether the echo power and leading-edge width (LEW) are included in the backscatter correction, the period of the backscatter correction, the period (epoch) over which elevation measurements are aggregated, and the radius of the triangulation scheme employed to fill data gaps. | | | | | | | | | | | | |

The choice of processing method had a significant impact on the spatial coverage of the elevation change product (Figure S3). For example, without spatial or temporal interpolation, the repeat track method surveyed on average 56% of the Antarctic ice sheet at monthly intervals when the measurements were gridded at 5 km spacing. At 140-day intervals, the majority (80%) of the ice sheet was sampled using the repeat track method, because areas episodically omitted at monthly intervals were significantly reduced. The remaining gaps were beyond the latitudinal limit of the satellite orbits, in areas of rugged terrain, and in northerly locations where the satellite ground tracks diverge. It is necessary to address the orbit divergence in particular, because ice sheet thinning tends to occur in coastal (and therefore northerly) locations that in turn tend to be under-sampled. To account for this, we performed a bilinear interpolation of elevation changes at each epoch using Delaunay triangulation. The radius of the triangulation scheme and the length of the time interval (epoch) over which elevation measurements were aggregated can each be varied to progressively reduce areas of omission. Among the scenarios we evaluate, the triangulation radius was varied between 0 and 150 km, and the length of the epoch was varied between 30 and 140 days. By comparison, the crossover method sampled only 11% of the grounded ice sheet, on average – a density that precludes application of the spatial interpolation scheme since the vast majority of grid cells were empty.

| 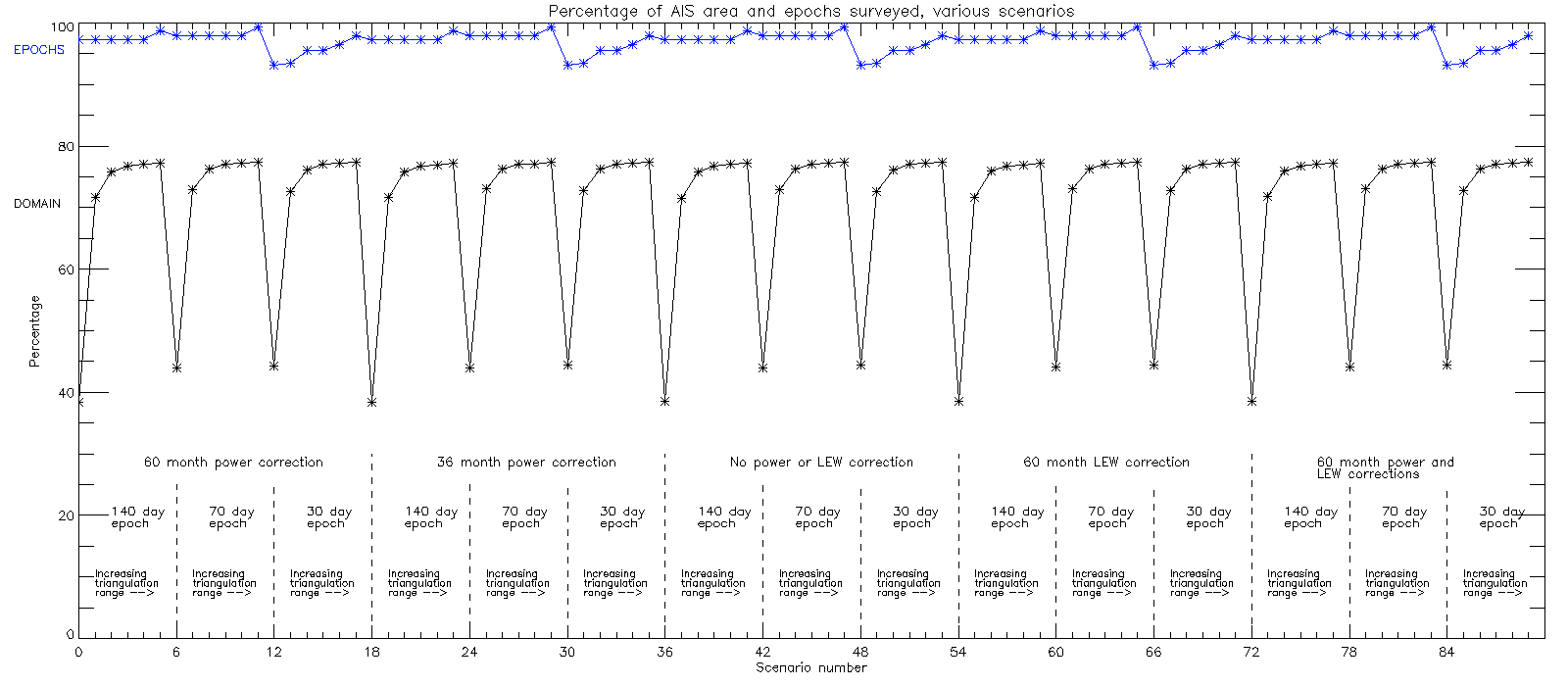 |
| --- |
| **Figure S3.** Proportion of the Antarctic ice sheet grid cells and epochs with valid elevation change measurements using the alternative data processing scenarios (see Table S1 for details). |

Other processing variations we considered included varying the period of time over which correlated fluctuations in elevation and backscattered power were determined and compensated for (we tested three options; 0-, 36-, and 60-month periods), and using a different approach to correct for radar penetration changes based on correlations between the echo leading edge widths and elevation. In most drainage basins, the impact of the processing scenarios we consider on the 25-year average rate of elevation change was slight (e.g. Figure 1), especially in East Antarctica where the signal was close to zero in all basins and under all scenarios. In West Antarctica there was a wider spread, especially in Amundsen Sea sector drainage basins (20, 21, and 22) where rates of surface lowering are relatively high. In these basins, the spread among scenarios is predominantly related to the spatial and temporal interpolation schemes. The largest diversity among scenarios was at the Antarctic Peninsula (basins 24-27), where the terrain is rugged and challenging for pulse limited altimetry in particular. Although we reported elevation changes in these regions from our optimal solution, we did not extend our analysis to report changes in mass due to the heightened sensitivity to processing approach; further work is required to establish the optimal solution.

**Text S5. Snowfall Variability**

Fluctuations in snow accumulation occur on a variety of timescales, and drive ice sheet surface elevation changes. In ice cores and climate model reanalyses, their impact diminishes with time, and it has been estimated, for example, that records longer than a decade are required to detect non-linear trends in ice sheet mass that are not meteorological in origin (Wouters et al., 2013). To assess their significance in relation to the observed elevation trends, we computed an estimate of expected snowfall variability in each drainage basin (Table 1).

According to an analysis of in-situ observations (Wingham et al., 1998), the 5-year variability of Antarctic accumulation fluctuations falls between 10 and 20 % of the long-term mean accumulation rate (MAR). This is three to four times larger than in regional climate models (Shepherd et al., 2012), and is indicative of the high variability that exists on spatial scales shorter than models are currently able to realise. As a conservative estimate, we used a value of 15 % - the mid-point 5-year snowfall variability within the in-situ records - as the basis of a scheme for estimating the variability at the scale of ice sheet drainage basins. Over regions that are large in comparison to the correlation scale of the altimeter measurements (~200 km), the temporal variability of accumulation fluctuations $\sigma_{A}$ was estimated to be

|  | $\sigma_{A}\sim\frac{1}{\rho_{A}}\sqrt{\frac{\overline{MAR}^{2}}{n}}0.15\sqrt{\frac{5}{T}}$ | Eq. (S4) |
| --- | --- | --- |

where $\rho_{A}$ is the density of the accumulation fluctuation, $\overline{MAR}^{2}$ is the spatial average of the mean accumulation rate (MAR) squared, $n$ is the effective number of independent values of MAR within the region taken to be $\sim A/{{\pi200}^{2}}$, and $T$ is the period in years of the accumulation fluctuation and $A$ is the region area in square kilometres. If $n<1$, we set it equal to unity, and we derived mean accumulation densities from a firn model (Ligtenberg et al., 2011) and rates from a regional atmospheric climate model RACMO2.3p2 (Melchior Van Wessem et al., 2018).

**Text S6. Firn Layer Thickness Change**

We used a semi-empirical firn densification model (Ligtenberg et al., 2011) driven by a regional climate model (Melchior Van Wessem et al., 2018) to simulate changes in the thickness of the Antarctic ice sheet firn layer. The firn model uses an improved expression for firn densification that is tuned to fit in situ depth-density observations, and is forced by temporal variations in the surface mass balance, surface temperature and near-surface wind speed as simulated by the RACMO2.3p2 regional climate model. The firn model covers the period 1979 to 2017 and is distributed on a 27 km grid with 10-day sampling. Using these data, we computed changes in the thickness of the firn layer across the ice sheet (e.g. Figure S4) and average rates of firn layer thickness change (e.g. Figure S5) during the period of our satellite altimeter record.

| 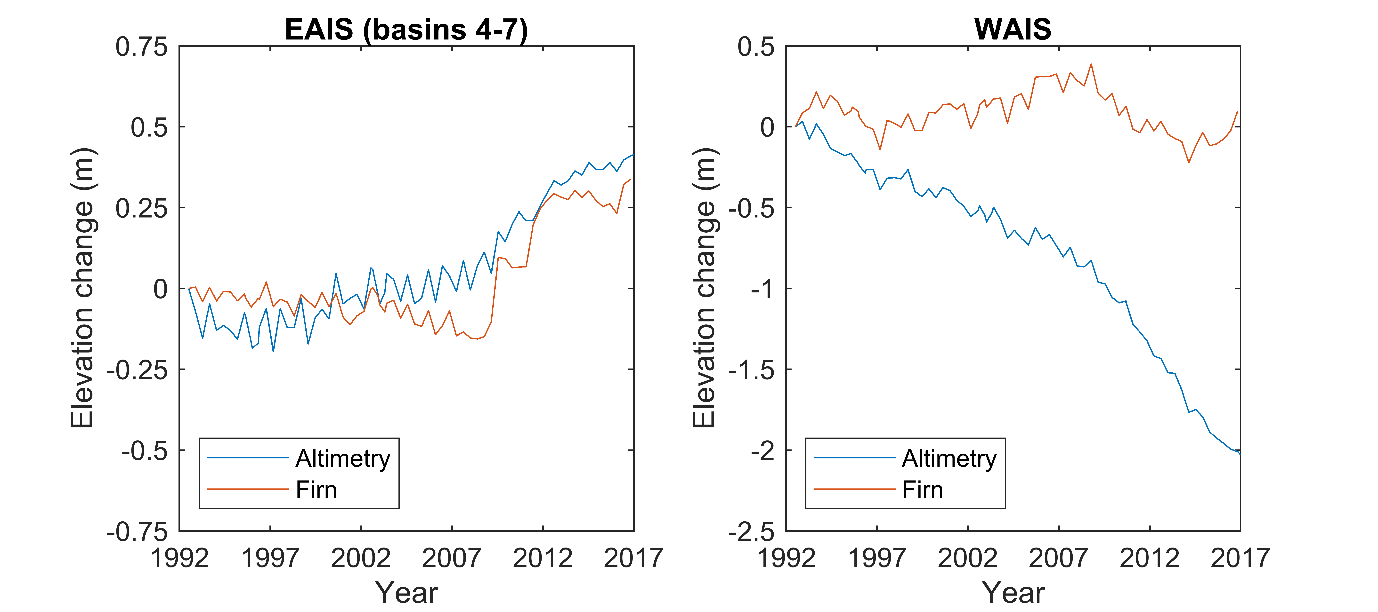 |
| --- |
| **Figure S4.** Simulated changes in firn layer thickness and observed changes in the surface elevation of (left) Dronning Maud Land and (right) West Antarctica. |

In East Antarctica and over the entire 25-year record, there is broad agreement between the model estimates of firn layer thickness change and the satellite measurements of surface elevation change (Figure S5), including modest thickening in coastal Dronning Maud Land, modest thinning in coastal Wilkes Land, and little change across the ice sheet interior. The Dronning Maud Land signal, in particular, has been associated with sharp increases in snowfall that occurred between 2009 and 2012 (Boening et al., 2012; Melchior Van Wessem et al., 2018), and is apparent in both the firn model and satellite altimeter data sets. Totten Glacier (basin 13) is, however, a notable exception; although there has been a 5 to 10 cm/yr reduction in firn thickness across the margins of the drainage sector, at 20 to 30 cm/yr the rate of ice sheet thinning is considerably larger and concentrated at the glacier, supporting independent evidence of dynamical imbalance (Li et al., 2015). At the Antarctic Peninsula, there are coherent patterns of ice sheet thickening and thinning in the southeast (basin 27) and southwest (basin 24), respectively; both signals are present within the modelled firn thickness change, suggesting the region is experiencing a significant meteorological imbalance in addition to localised glacier speedup (Hogg et al., 2017; Rignot et al., 2004; Rignot et al., 2005; Scambos et al., 2004). In West Antarctica, however, there is strong departure between the patterns of elevation and firn thickness change, as a consequence of the widespread dynamical imbalance that has occurred in the Amundsen Sea (Rignot et al., 2008) and Siple Coast (Joughin and Tulaczyk, 2002) sectors. Although the region has experience a relatively large fluctuation in snowfall (Melchior Van Wessem et al., 2018), the resulting changes in firn layer thickness (Figure S4) are small in comparison to the wider pattern of surface elevation change driven by glacier thinning.

| 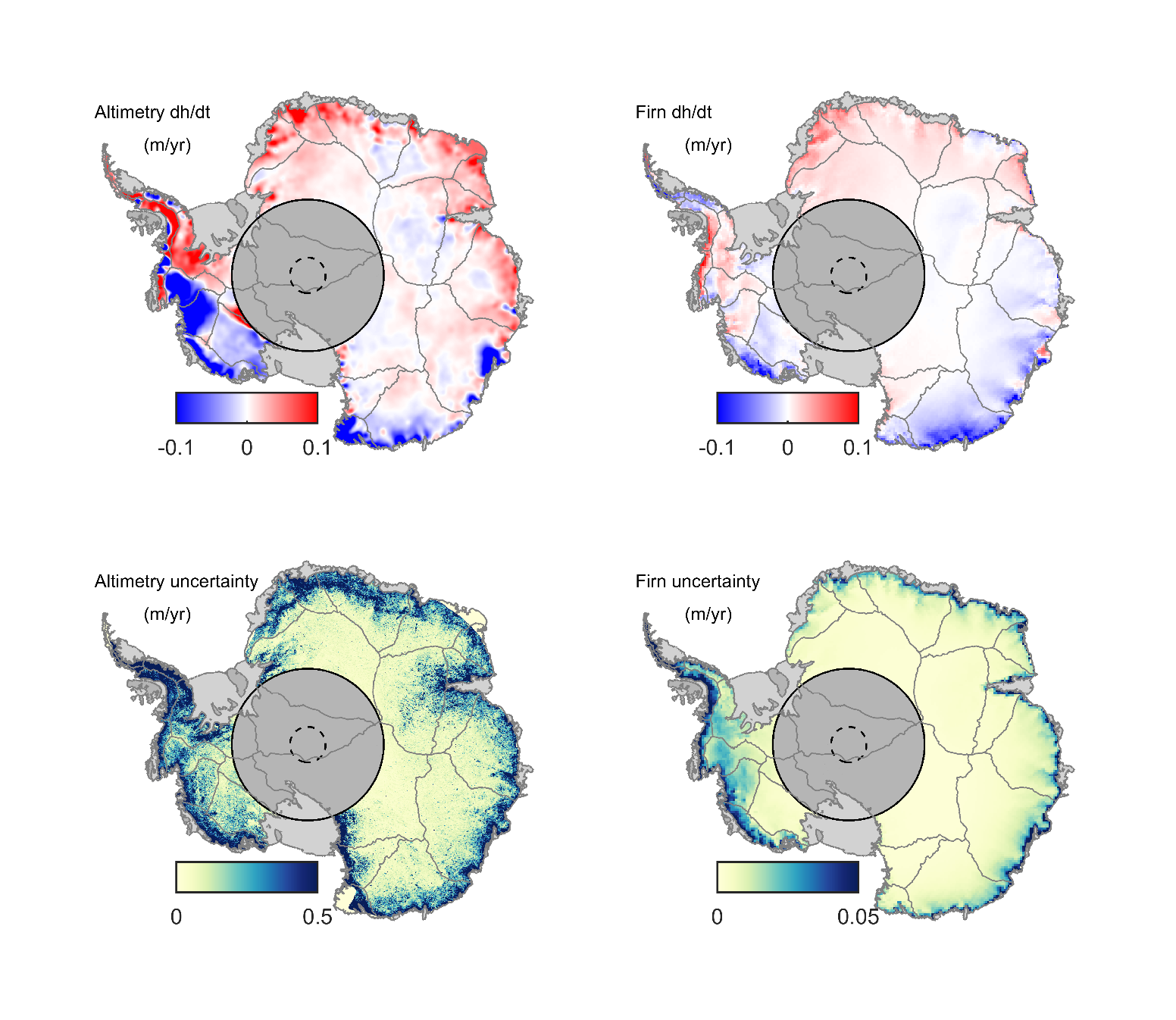 |
| --- |
| **Figure S5.** Average rate of ice sheet elevation change (left) and firn layer thickness change (right) over the period 1992 to 2017. |

Although there are qualitative similarities, the level of agreement between the modelled firn layer thickness changes and the observed surface elevation changes deteriorates over shorter temporal and spatial scales. For example, although the magnitude and approximate timing of the Dronning Maud Land snowfall increase are comparable in the model and satellite data (Figure S4), the event is more abrupt according to the firn model and so rates of change differ markedly. Over decadal (Wingham et al., 2006a) and seasonal (Ligtenberg et al., 2011) timescales, firn thickness and surface elevation cycles have also been shown to be less well matched. At local scale, there is often poor correlation between changes in firn layer thickness and surface elevation as the signals are seldom coincident in space, as a consequence of uncertainties in the firn model and satellite data and also due to localised processes affecting the ice sheet that are not meteorological in origin – for example fluctuations in subglacial lake hydrology (Wingham et al., 2006b). To assess the degree of similarity, we correlated trends in firn layer thickness and surface elevation at various spatial and temporal scales in East Antarctica, where few signals of ice dynamical imbalance have been reported (Table S2). In this comparison, the firn model and satellite elevation changes are most strongly correlated over the full 25-year period and at the scale of drainage basins (r^2^=0.48), but the degree of similarity drops significantly at shorter spatial (r^2^<0.2) and temporal (r^2^=0.3) scales. Care should therefore be exercised when considering model estimates of firn layer thickness change as a correction to satellite radar altimeter data in Antarctica.

|  | Grid-scale | | Basin-scale | |
| --- | --- | --- | --- | --- |
|  | 5-year | 25-year | 5-year | 25-year |
| EAIS | 0.16 | 0.18 | 0.30 | 0.48 |
| **Table S2.** Average correlation between time series of modelled firn thickness change and observed surface elevation change computed at either the 5 km grid scale of the satellite data or the scale of ice sheet drainage basins (see Figure 2) and over five successive 5-year and one 25-year intervals. | | | | |

**Text S7. Locating Areas of Ice Dynamical Imbalance**

We identified areas of ice dynamical imbalance as regions of large and persistent elevation change that were significantly (>0.1 m/yr) different to firn thickness change during the same period, taking their respective uncertainties into account, and after fitting time-dependent quadratic polynomials to the time series at each grid cell (Figure S6). Firn thickness changes were determined from a firn density model (Ligtenberg et al., 2011) driven by the RACMO2.3p2 regional climate model (Melchior Van Wessem et al., 2018). The full 25-year period of the satellite altimeter data record was used, except in the area south of the ERS-1, ERS-2 and ENVISAT orbital limits, where we used the 7-year period of CryoSat-2. The initial classification was then edited using a sequence of morphological closing and opening operations within 5 x 5 regions to homogenise patches of dynamic thinning or thickening; opening operations carry out erosion followed by dilation and closing operations do the reverse. We removed all connected patches if they contain fewer than 100 grid cells. The scheme resolved areas of dynamic thickening and thinning.

Because ice speedup in Antarctica has in places spread inland over sub-decadal timescales (Hogg et al., 2017; Joughin et al., 2003; Mouginot et al., 2014; Rignot et al., 2005), we allowe areas of dynamic imbalance to evolve through the altimeter record if there is clear evidence to support this; otherwise they remained static in shape. To do this, we used the quadratic fit to the firn-corrected altimeter elevation change time series in areas of identified dynamical imbalance to identify large (>750 pixel) regions of significant (>0.08 m yr^-2^) accelerated elevation change. According to this scheme, only two regions exhibited significant acceleration in the rate of ice thickness change – basins 21 and 22. In these basins, we allowed the area of dynamical imbalance to evolve over time by computing the date on which the elevation trend at individual grid cells exceeds the firn thickness trend, again considering their respective uncertainties.

| 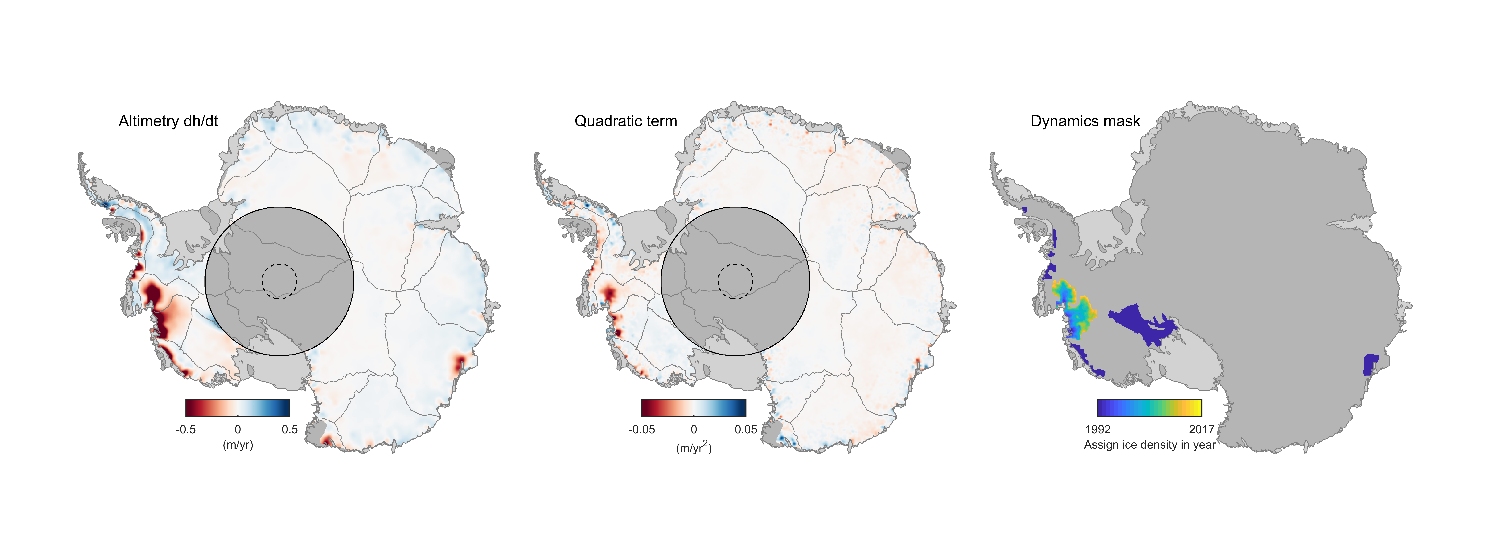 |
| --- |
| **Figure S6.** (left) Average rate of Antarctic ice sheet surface elevation change between 1992 and 2017 after accounting for changes in firn layer thickness determined from a second-order polynomial fit to the data, and (centre) the quadratic coefficient from the polynomial fit which highlights regions where a significant change in elevation rate has occurred during the 25-year survey. Also shown (right) is the locus and date on which areas are first identified to be in a state of ice dynamical imbalance, taking the average change and acceleration into account (see text for details). |

Altogether, we identified dynamic thickening in two drainage basins and dynamic thinning in eight others (Figure S7). The area of thinning evolves in the Pine Island and Thwaites glacier drainage basins, consistent with a previous assessment (Konrad et al., 2017), increasing from 1,125 km^2^ in 1992 to 200,700 km^2^ in 2017 which corresponds to a 150-300 m/yr reduction in the average flow speed of dynamically thinning ice over the survey period, or a 200-350 km inland propagation.

| 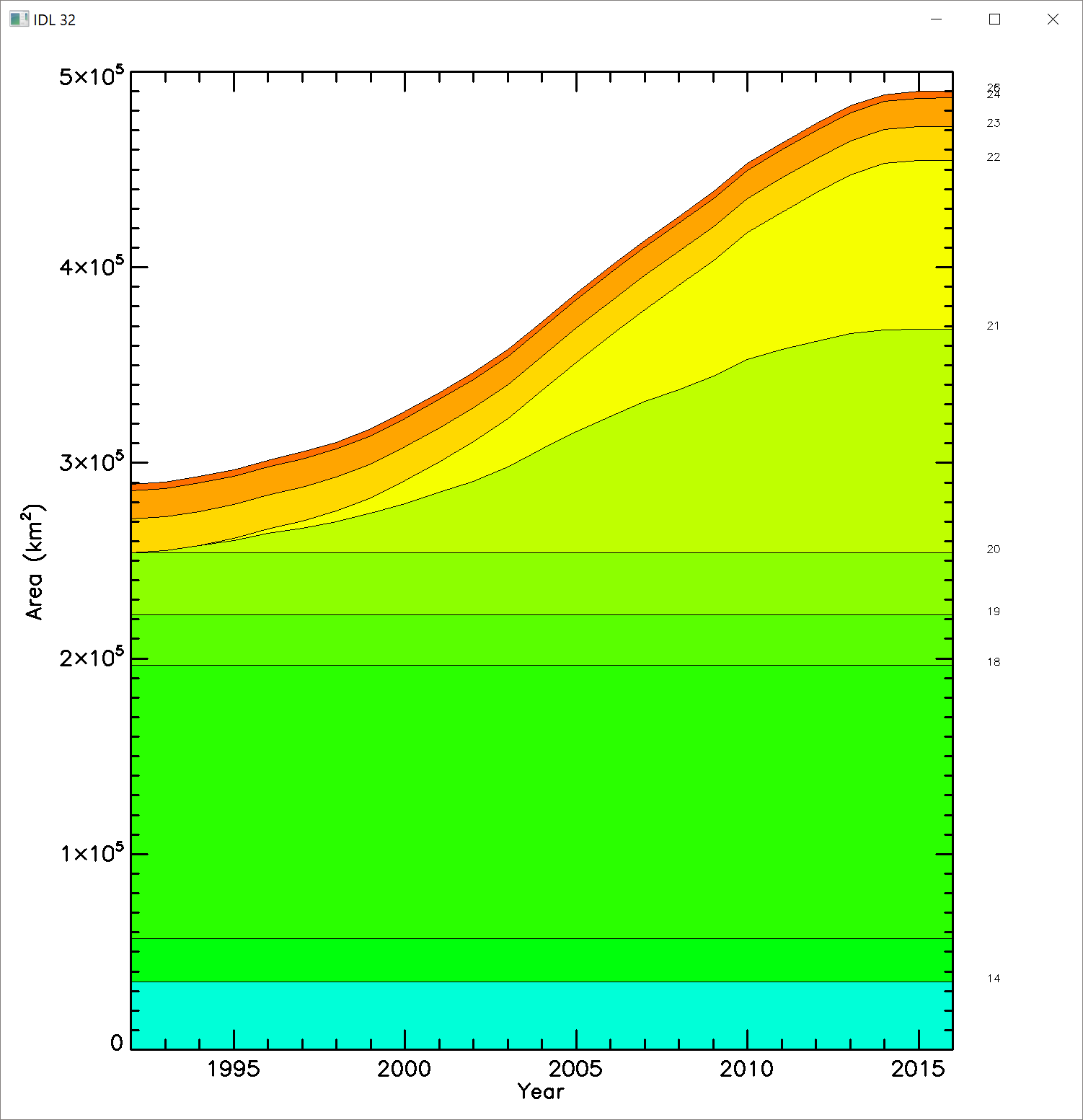 |
| --- |
| **Figure S7.** Area of Antarctic drainage basins (see Figure 2) identified to be thinning, dynamically, at dates during the altimeter record. In basins 21 and 22, the area of dynamical imbalance evolves over time (see text for details). |

**Text S8. Computing Ice Sheet Mass Trends**

To estimate trends in the mass balance of East and West Antarctic drainage basins (Figure S8 and Table 1) and in areas of ice dynamical imbalance (Table S3), we used our empirical discrimination of ice dynamical imbalance (see Figure S6) to classify elevation changes occurring at the densities of snow and ice. We used a value of 917 kg/m^3^ for the ice density, and spatially varying snow densities derived from a firn densificaton model (Ligtenberg et al., 2011) applied to regional climate model output (Melchior Van Wessem et al., 2018). Although the firn densities ranged from 300 kg/m^3^ in the ice sheet interior to up to 500 kg/m^3^ towards the coast (Figure S8), their impact on basin-scale and ice-sheet wide estimates of masss trends is nevertheless small (<1 Gt/yr) when compared to estimates derived using a constant density of 350 kg/m^3^ (Figure S9). The optimal solution most closely matches GRACE (see also section 9.3). At ~500 Gt over 3 to 4 years, the estimated change in mass of East Antarctica during the early 1990’s is anomalously high and, although it falls at the upper range of variability estimated in regional climate models (Shepherd et al., 2012), the signal is relatively poorly resolved, owing to interruptions in the temporal sampling of elevation during the early part of the ERS-1 mission.

| 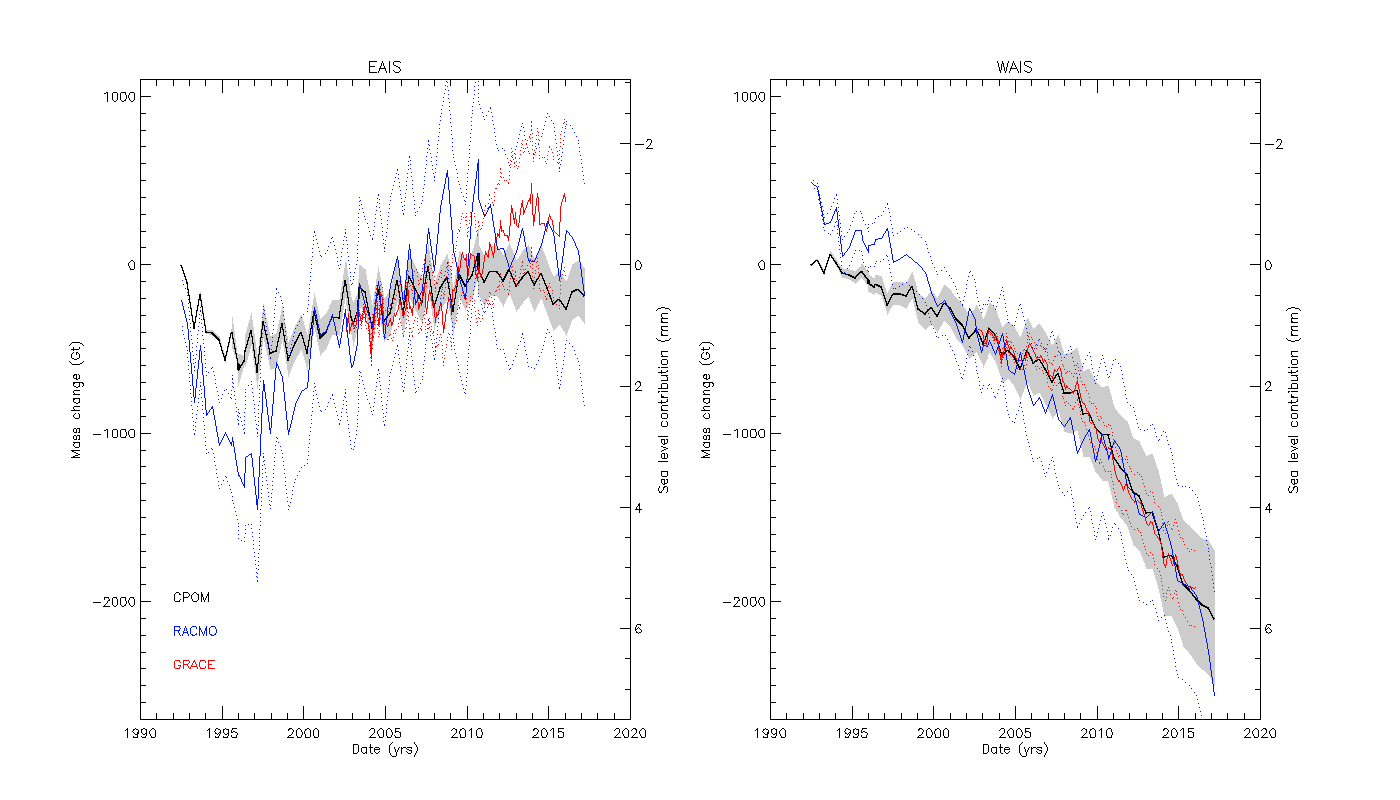 |
| --- |
| **Figure S8.** Mass change in East (left) and West (right) Antarctica as determined from satellite altimetry using a classification of areas in a state of ice dynamical imbalance (the optimal solution, black line) and the estimated 1-sigma (67%) uncertainty (grey shaded area). Also shown for comparison are mass changes determined from an alternative altimetry solution directly employing a firn density model and a regional climate model (blue) and from GRACE (red). |

Although this approach has been shown to produce estimates of ice sheet mass balance that are consistent with results derived from other geodetic techniques (Shepherd et al., 2018; Shepherd et al., 2012), it does not account for potentially coincident signals of meteorological and dynamical imbalance, and this introduces additional error into mass trends calculated in this way. To account for this additional error, we treated the expected variability in snowfall as an equivalent source of elevation change uncertainty in our mass trend solutions. Specifically, we estimated the total uncertainty on measured elevation changes by summing in quadrature the uncertainties associated with our satellite measurements together with the snowfall variability uncertainty. We then converted these to an equivalent mass change uncertainty, using a density of either snow or ice, based upon our empirical discrimination of ice dynamical imbalance. Where basins were a combination of areas classified as snow and ice, the respective uncertainties associated with each sub region were summed in quadrature to determine the total basin uncertainty. As with our estimates of elevation change, we computed the uncertainty on the mass trends by dividing the total mass uncertainty accumulated by the end of the time series by the duration of the record.

|  | **1992-1997** | **1997-2002** | **2002-2007** | **2007-2012** | **2012-2017** | **1992 - 2017** |
| --- | --- | --- | --- | --- | --- | --- |
| Pine Island Glacier | -2 ± 1 | -10 ± 4 | -18 ± 9 | -48 ± 10 | -55 ± 4 | -26 ± 13 |
| Thwaites Glacier | -12 ± 1 | -25 ± 4 | -37 ± 11 | -58 ± 11 | -76 ± 6 | -41 ± 16 |
| Totten Glacier | -10 ± 1 | -6 ± 1 | -10 ± 1 | -11 ± 1 | -13 ± 1 | -9 ± 2 |
| Siple Coast | 26 ± 5 | 4 ± 1 | 20 ± 2 | 11 ± 1 | 16 ± 1 | 13 ± 4 |
| Getz Ice Shelf | -7 ± 3 | -6 ± 3 | -15 ± 3 | -24 ± 3 | -27 ± 3 | -14 ± 6 |
| Bellingshausen Sea | -8 ± 1 | -3 ± 1 | -7 ± 1 | -6 ± 1 | -11 ± 1 | -6 ± 3 |
| Total | -13 ± 13 | -45 ± 13 | -69 ± 26 | -137 ± 26 | -166 ± 16 | -84 ± 44 |
| **Table S3.** Average mass balance of Antarctic drainage basin sections identified to be in a state of dynamical imbalance over successive 5-year intervals, in Gt/yr**.** | | | | | | |

As an alternative approach, we also computed trends in ice sheet mass using the satellite altimeter elevation changes and model estimates of surface mass balance and firn layer thickness change. First, we used monthly output from a regional atmospheric climate model (RACMOv.2.3p2) (Melchior Van Wessem et al., 2018) to estimate fluctuations in surface mass balance alone. We then used monthly output from a firn densification model (Ligtenberg et al., 2011) to adjust the satellite-detected ice sheet elevation changes for the associated signal and, from these data, estimated fluctuations in ice mass alone. The sum of these two terms is then our alternative estimate of the overall ice sheet mass trend. Prior to use, the surface mass balance and firn densification model datasets were resampled from their original 27 km square resolution to match the 5 km square grid of the satellite altimeter time series. The firn densification model data were also only available for the period between 1979 and 2016, which prevents us from using this approach across the final year of the altimeter data. We estimated the uncertainty on mass trends computed in this way as the sum of contributions due to errors in the modelled firn elevation change, in the satellite elevation change, and in the surface mass balance. Although the firn-correction approach does account explicitly for the impact of snowfall fluctuations, these are not always well matched with satellite radar altimeter elevation changes (Horwath et al., 2012; Wingham et al., 2009), and compensating for them also leads to relatively large uncertainties and large residual signals of ice imbalance. Possible reasons for the mismatch include variations in the radar altimeter observations due to signal penetration into the snowpack and the manner in which these penetration signals are corrected, uncertainties in model simulations of surface mass balance, and uncertainties in the parameterisation of firn compaction.

| 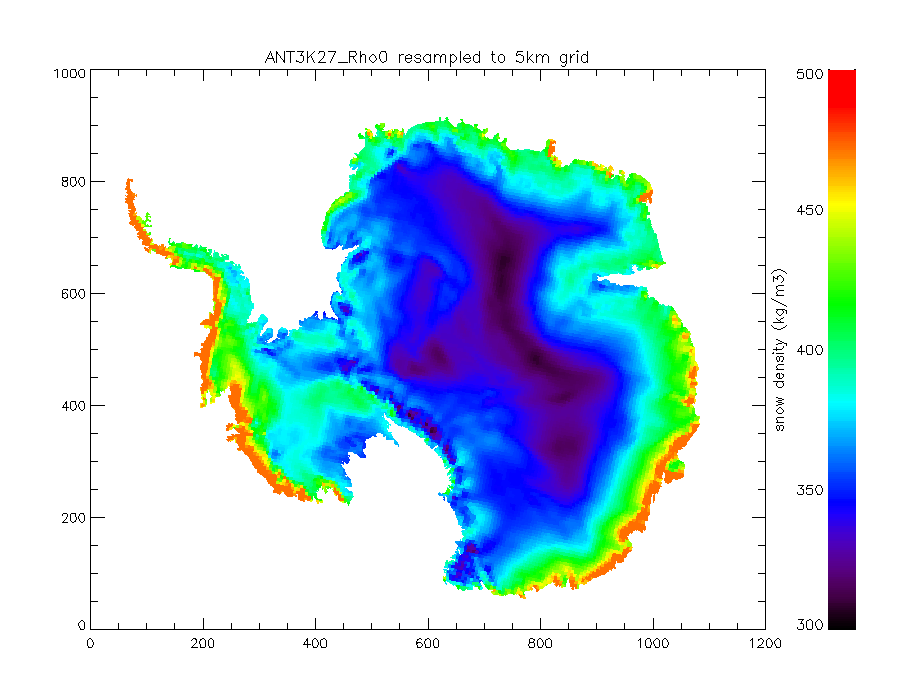 | 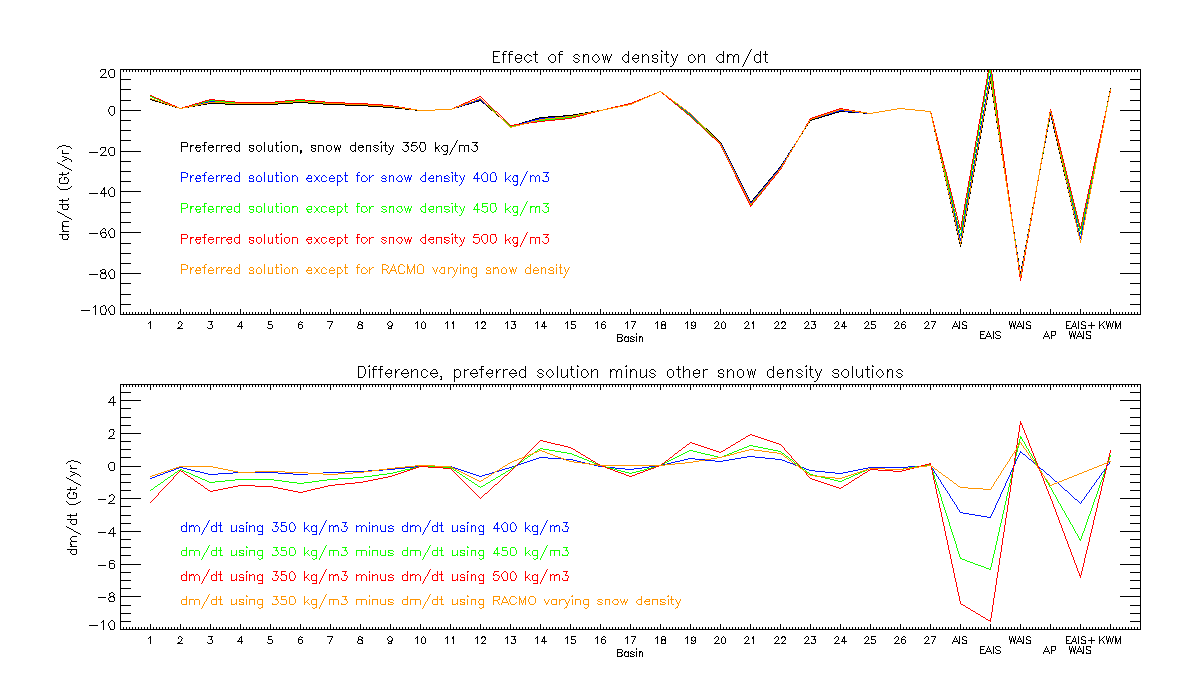 |
| --- | --- |
| **Figure S9.** Spatially variable average firn density (left) derived from a firn density model driven by a regional climate model and its impact on basin-wide estimates of ice sheet mass balance (right) relative to estimates computed using a constant firn density. | |

**Text S9. Evaluating Ice Sheet Elevation and Mass Trends**

We evaluated our ice sheet elevation and mass change solutions by considering their ability to (i) address under-sampling of signal at northerly latitudes due to gaps between the satellite orbit ground tracks, (ii) match independent estimates of elevation change derived from airborne laser altimetry, and (iii) match independent estimates of ice sheet mass change derived from satellite gravimetry.

**Text S9.1 Evaluation of Under-Sampling**

Of the 90 alternative elevation change solutions we have developed, one group was designed to assess the impact of signal under-sampling. This was achieved either by varying the degree of spatial (0 to 30 km) or temporal (30 to 140 days) interpolation. We also tested separately the use of an elevation change solution determined at orbit ground track crossing points (crossovers). As an indication of their sensitivity, we computed the average elevation change of East and West Antarctica using all 90 scenarios (Figure S10). When gridded at 5 km, crossover solutions were based on measurements from a small (11%) fraction of the ice sheet and, consequently, resulted in average elevation changes that were strongly dependent on the degree of interpolation. In contrast, plane fit solutions were based on measurements from the majority (56%) of the ice sheet, leading to more stable solutions requiring less interpolation. For example, in East Antarctica the average elevation rate of plane fit solutions changes little according to the scenario tested, fluctuating in the range 0.4 and 0.7 m/yr. However, in West Antarctica there is a tenfold greater spread among the various plane fit scenarios we have tested due to the high concentration of thinning in northerly locations, where the satellite orbits diverge. The impact of under-sampling northerly areas is progressively reduced in both ice sheets as the degree of spatial or temporal interpolation increases, ultimately leading to stable solutions. This leads us to prefer scenarios that (i) use the plane fit elevation change solver rather than crossovers, (ii) average measurements in 140-day epochs, and (iii) interpolate elevation changes over distances of 20 km to fill any remaining gaps.

| 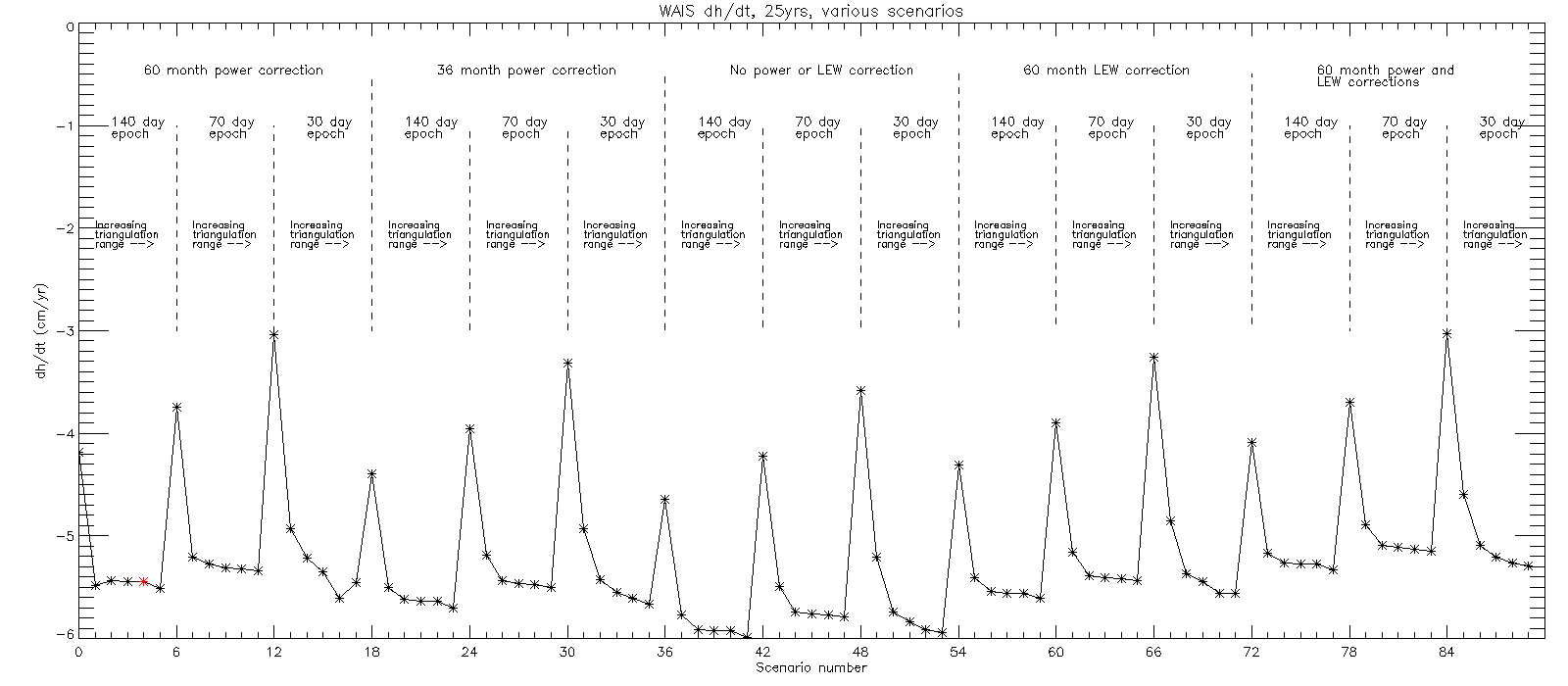 |
| --- |
| 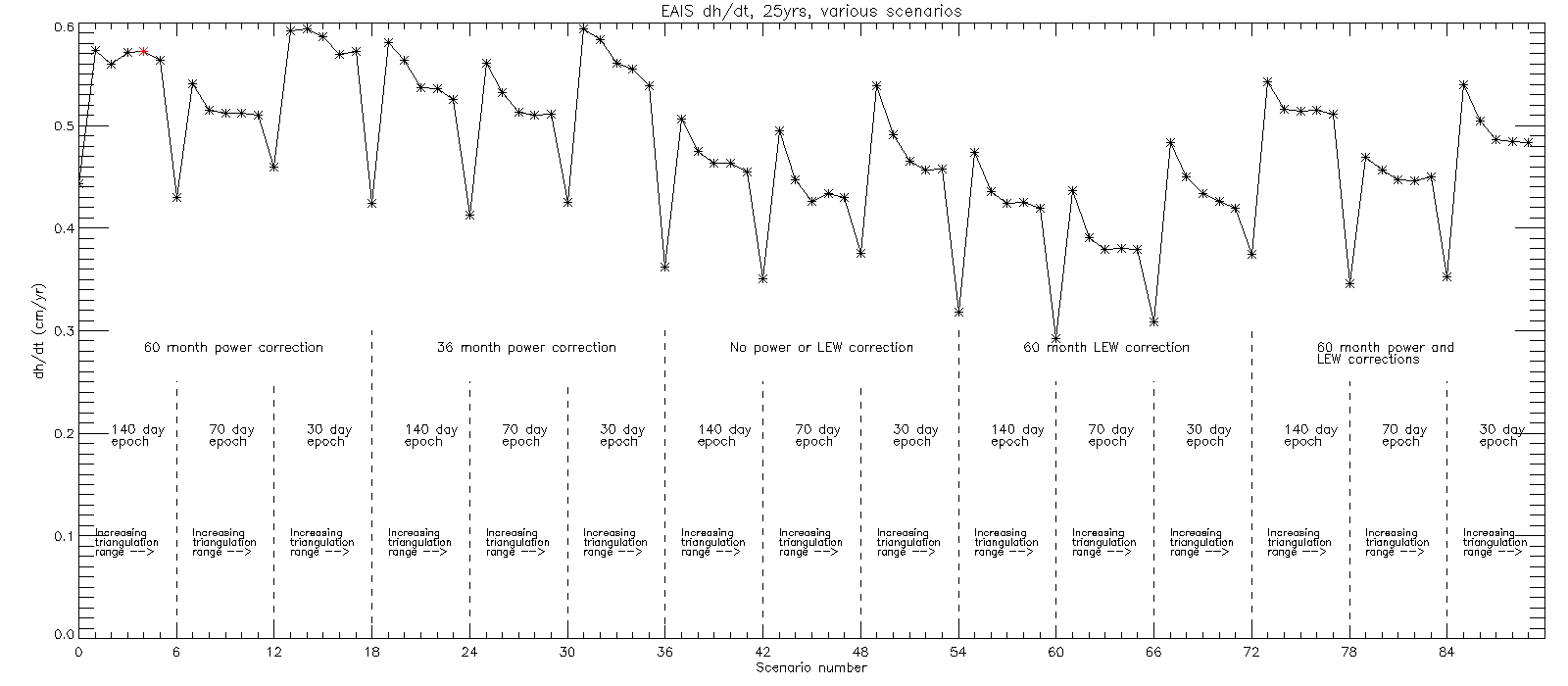 |
| **Figure S10.** Average rate of elevation change in West (top) and East (bottom) Antarctica derived from the 44 data processing scenarios (see Table S1 for details) between 1992 and 2017. The optimal scenario (scenario 4) is shown in red. |

**Text S9.2 Comparison to Airborne Elevation Changes**

As a next evaluation step, we compared the satellite altimeter elevation change time-series developed from the processing scenarios to independent estimates derived from airborne laser altimeter measurements (Studinger, 2014). The airborne dataset included 830,008 individual measurements of elevation change computed from Operation IceBridge Airborne Topographic Mapper surveys acquired between 2002 and 2017 (see Figure S11 for locations). Of these measurements, 544,422 (66%) coincided with locations present within our satellite elevation change time series; the remainder are generally in regions of rugged terrain such as the Antarctic Peninsula, where the satellite data set is incomplete.

The satellites used in our study operate at similar microwave frequencies, and so differences in radar penetration are not anticipated over equivalent targets. However, there is a factor ~10 improvement in the spatial resolution of synthetic aperture radar interferometric (SIN) altimetry (employed by CryoSat-2 in coastal regions) relative to pulse-limited altimetry (employed by CryoSat-2 elsewhere and by ERS and ENVISAT everywhere), and any differences in surface roughness within the illuminated target would impact on the range-distribution of scatterers (Arthern and Wingham, 1998; Brown, 1977). Although the two effects are not the same, similar approaches are employed to address their variability – namely retracking the radar waveforms (Bamber, 1994; Davis, 1996, 1997; Helm et al., 2014) and applying further corrections to account for residual correlated changes in echo shape and retrieved height (Davis and Ferguson, 2004; Khvorostovsky, 2012; Wingham et al., 1998). We therefore separated, in our comparison, elevation changes determined by CryoSat-2 operating in high-resolution SIN model from measurements acquired by CryoSat-2 and other missions in pulse-limited, low-resolution model (LRM).

| 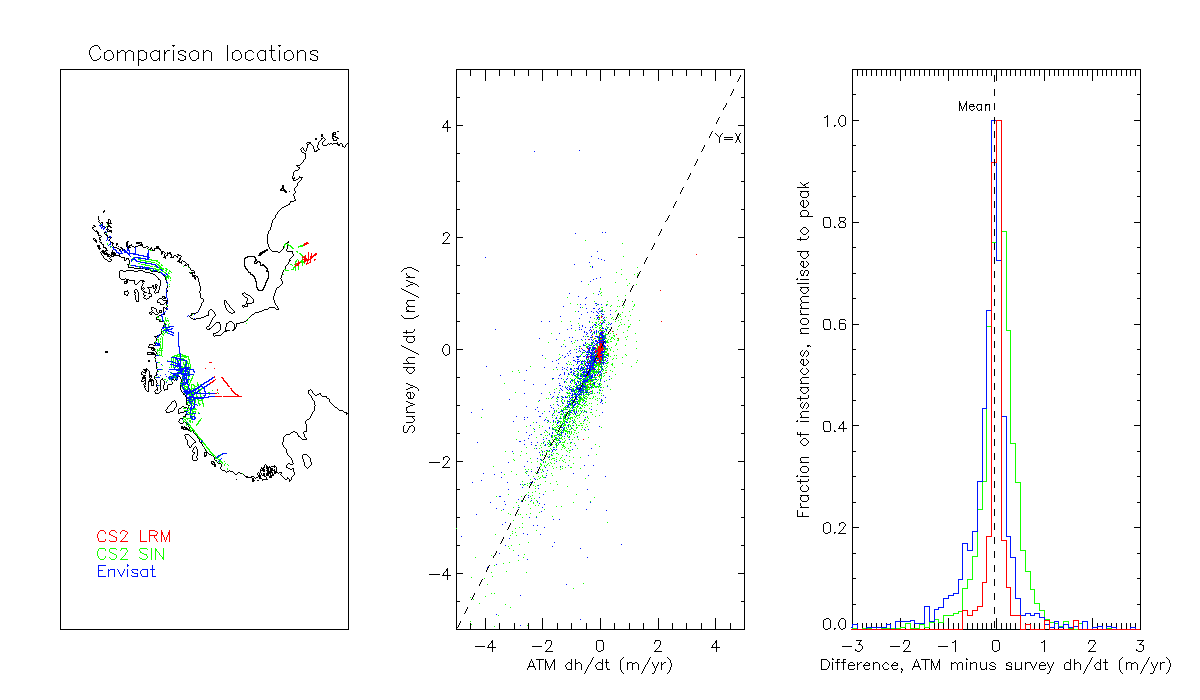 |
| --- |
| **Figure S11.** A comparison of airborne and satellite derived elevation changes, including the locations of the airborne data (left), a scatter plot of elevation rates computed from each data set using the optimal scenario (centre), and a histogram illustrating the distribution of differences between the two data sets using the optimal scenario (right). Elevation data recorded in low resolution model (LRM) are highlighted in red (CryoSat-2) and blue (ENVISAT), elevation data recorded in high-resolution synthetic aperture radar interferometry (SIN) mode are highlighted in green. |

Although the satellite data are regularly gridded, the airborne data are recorded with 20 times finer spatial resolution (250 m) along flight lines which preferentially sample ice that is thinning fastest. This irregular sampling pattern leads to measurements that tend to be biased high, on average, when compared to coarsely gridded data (Flament and Remy, 2012; McMillan et al., 2014). We estimated the airborne elevation rates to be biased high by 8% (or 5 cm/yr, on average), by considering their sampling of a high-resolution ice velocity map (Rignot et al., 2011) at point locations relative to the 5 km satellite grid.

We averaged rates of elevation change computed from pairs of IceBridge passes in 5 km grid cells to match the spatial resolution of the satellite data. This comparison was restricted to 2712 grid cells common to both datasets, where the root mean square error and standard deviations of the airborne and satellite estimates were less than 0.4 m/yr, and where the airborne data spanned more than 2 years. For each of the processing scenarios, we computed the mean and standard deviation of the difference between the bias-adjusted airborne elevation rates and the satellite elevation rates (Figure S12). Across all scenarios, the average difference between the adjusted airborne and satellite measurements of elevation change fell in the range ± 5 cm/yr. However, the largest average departures by far are among solutions that employed alternative approaches to correct for fluctuations in backscattered power. Specifically, the degree of departure increased as the duration of the period used to perform power corrections decreased from 60 to 0 months, and also with the use of the echo leading edge width as an additional factor which in every case led to poorer agreement. Aside from these outlier scenarios, the average difference between the airborne and satellite elevation changes was less than 1 mm/yr. The standard deviation between the gridded satellite and airborne elevation changes fell between 30 and 50 cm/yr in all scenarios, with again the greater departure occurring for those that vary the power correction approach. This spread is comparable to the estimated certainty of the airborne and satellite elevation differences. Of the grid cells where the satellite and airborne data coincide, 2703 were recorded in SIN model (only CryoSat-2) and 2006 were recorded in LRM (some CryoSat-2 and ENVISAT), and the average differences were +4 ± 53 and -16 ± 61 cm/yr, respectively. Overall, the LRM data sampled a similar range of signals to the SARIn data (see Figure S11), though both their spread and difference relative to the airborne data were slightly higher. Although this finding is consistent with their expected poorer performance, the difference is much smaller than our estimated uncertainty of individual time series. The level of agreement between the various satellite elevation change scenarios and the airborne data leads us to identify solutions that (i) do not apply a leading edge width correction, and (ii) use a 60 month period as the basis of corrections to compensate for correlated fluctuations in elevation and backscattered power as being optimal.

| 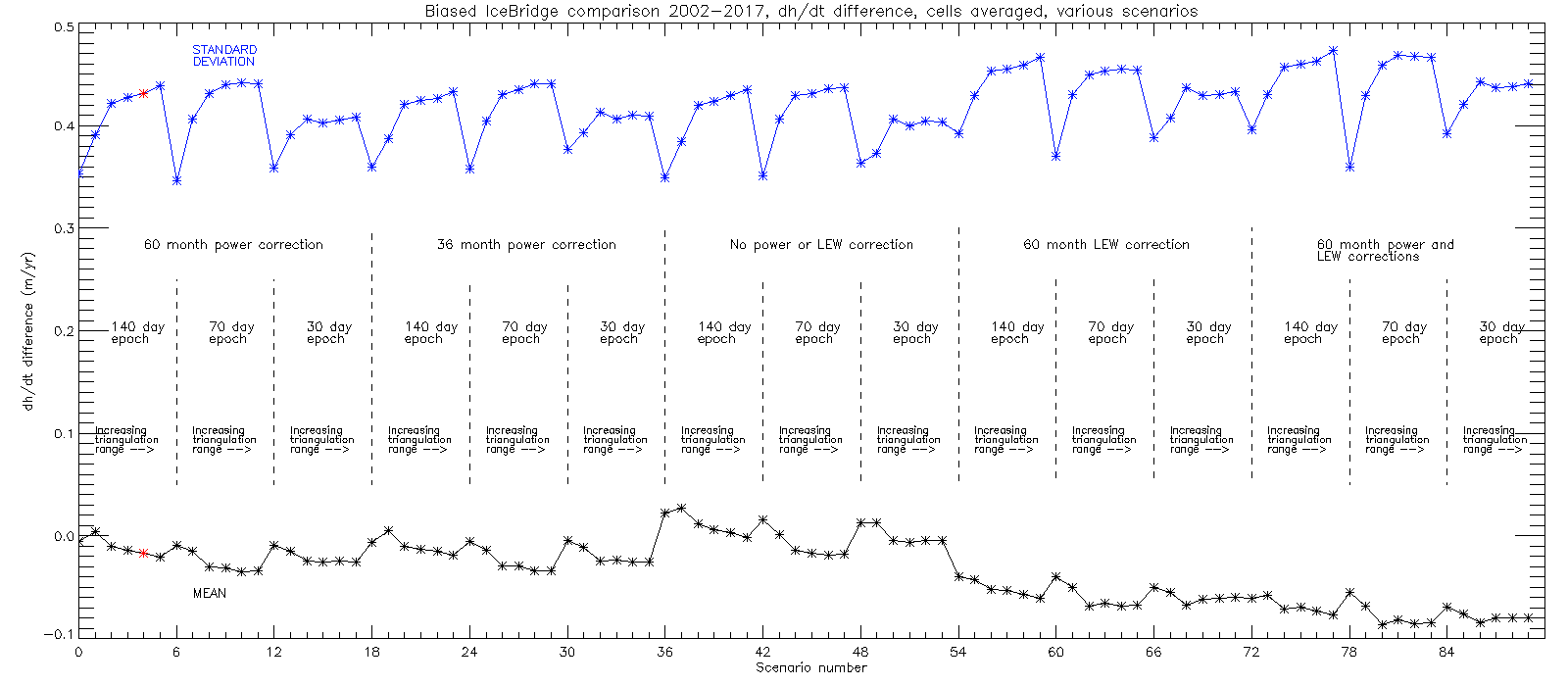 |
| --- |
| **Figure S12.** A comparison of airborne and satellite derived elevation changes, including the average and standard deviation of their differences from the satellite data at common locations across all repeat track processing scenarios. |

**Text S9.3 Comparison to GRACE Mass Changes**

As a final evaluation step, we compared ice sheet mass trends developed from the 90 alternative elevation change scenarios and two mass balance approaches to independent estimates derived from measurements of changing gravitational attraction acquired by the Gravity Recovery and Climate Experiment (GRACE) mission (Groh and horwath, 2016).

We computed drainage basin mass balance from the altimeter data in two ways; first using our discrimination of ice dynamical imbalance to separate fluctuations in snow and ice mass, and then using the direct application of a firn compaction model (Ligtenberg et al., 2011) and regional climate model (Melchior Van Wessem et al., 2018). We did not calculate mass balance from the crossover solutions, as their sparse sampling is not well suited to the regional integration. The GRACE data are time-series of estimated mass changes over the period 2002.624 to 2016.042 posted on a polar stereographic projection with a grid resolution of 50 km. We integrated the GRACE mass changes across each drainage basin by resampling them to the same 5 km resolution as the altimeter time series using a nearest-neighbour scheme and summing the resulting data within each boundary. We then computed rates of mass change from the GRACE and altimeter data using linear fits to the basin-wide integrated mass changes over the 13.4-year period of the GRACE measurements.

Comparison between altimetry and GRACE estimates of mass balance were performed at the scale of ice sheet drainage basins and on time series with seasonal cycles removed to limit the impact of mismatches in their amplitude within the altimetry and firn model data (Ligtenberg et al., 2011). We also compared estimates of specific mass balance (rates of mass change per unit area) to remove the potential impact of the drainage basin area, which may alter the correlation between the independent measurements as it is a common factor. We were not able to perform the comparison in drainage basins 25 and 26 situated at the northern tip of the Antarctic Peninsula, as they were poorly sampled and resolved in the altimetry and GRACE measurements, respectively. In all, we were able to compare mass balance and specific mass balance estimates in 25 drainage basins; all of East and West Antarctica, and the southern portion of the Antarctic Peninsula.

| 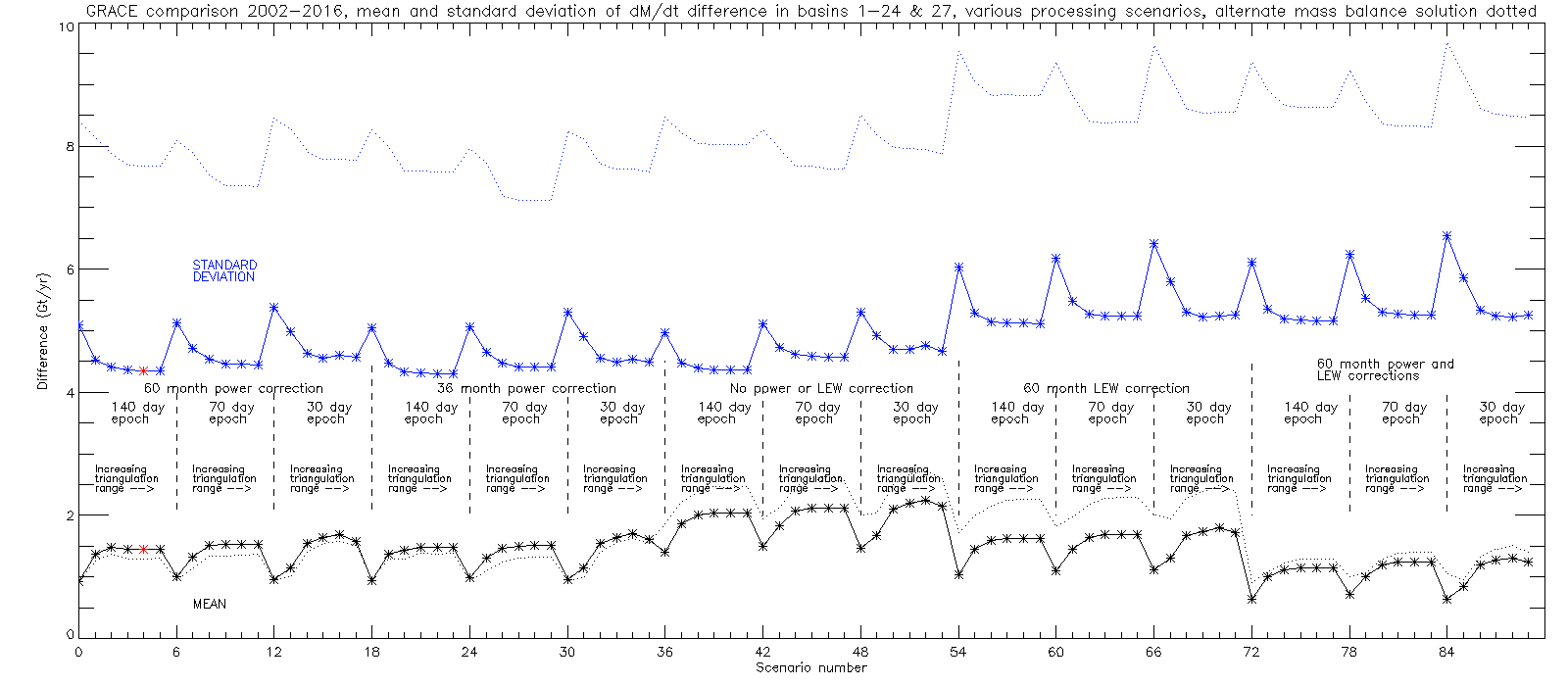 |
| --- |
| 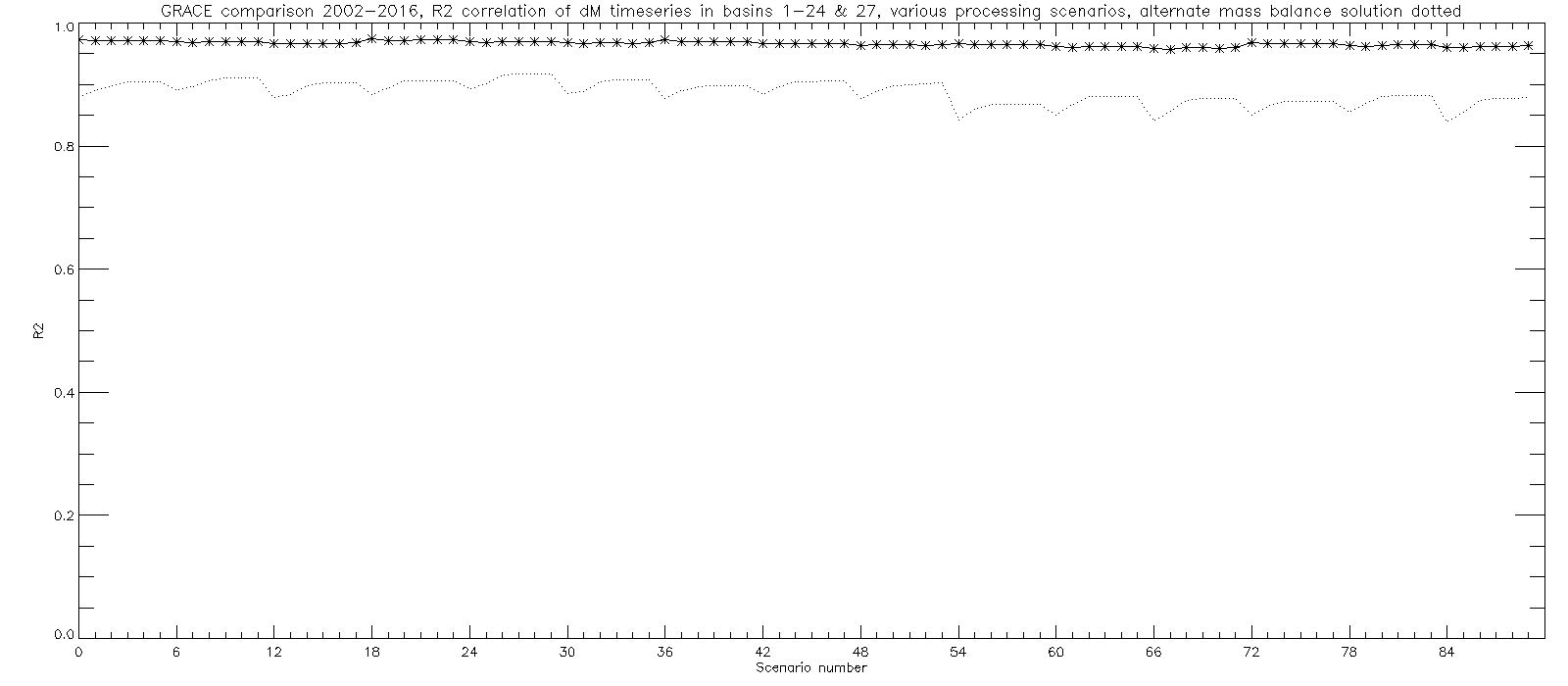 |
| **Figure S13.** The average difference and standard deviation (top) and correlation coefficient (bottom) between estimates of mass trends determined within 25 Antarctic ice sheet drainage basins from GRACE satellite gravimetry and from our satellite altimetry data. Altimetry mass trends were determined using either a classification of signals occurring at the density of snow and ice (solid lines) or using explicit corrections for changes in firn layer thickness and surface mass balance (dotted lines). |

When employing our discrimination of ice dynamical imbalance to estimate mass balance, there was generally very good agreement with mass trends estimated by GRACE at the scale of drainage basins (Figure S13, Figure S14, and Table S4). Across the 25 basins common to both datasets, the average difference between the altimetry and GRACE mass balance estimates falls in the range 1 to 2 Gt/yr, the correlation coefficient is everywhere very high (r^2^>0.95 ), and the standard deviation of the differences falls in the range 4 to 6 Gt/yr across all scenarios. The correlation coefficient is equally high when specific mass balance is compared (Table S3), suggesting that the comparison is not biased by the drainage basin area (a factor in common). The largest differences occur in Dronning Maud Land (basins 6 and 7), where a large recent accumulation fluctuation occurred between 2009 and 2011 (Boening et al., 2012), and in the Amundsen Sea sector, where the coarse resolution of the GRACE measurements allows mass losses from Thwaites Glacier (basin 21) to leak across the western drainage divide into the adjacent basin (basin 20). In general, increasing the degree of spatial and temporal interpolation leads to a reduction in the variance between the GRACE and altimeter data, especially in West Antarctica where thinning is concentrated in gaps between the satellite altimeter ground tracks.

Although the degree of correlation and mean difference between mass balance solutions developed from GRACE and the altimetry solution explicitly using firn (Ligtenberg et al., 2011) and surface mass balance models (Melchior Van Wessem et al., 2018) were comparably low (Table S4), the spread (standard deviation, s.d.) of the data is significantly (70%) higher (7.6 Gt/yr v 4.5 Gt/yr)(Figure S14). In the case of specific mass balance, while the degree of correlation and spread of the two alternative altimetry solutions relative to GRACE are comparable, their mean differences are not; the firn and climate model solution differs by 2.4 kg/m^2^/yr, on average, whereas the ice dynamical imbalance classification scheme approach differs by 0.1 kg/m^2^/yr, on average. On the basis of this comparison, we identify the mass balance solution implementing the classification scheme as the optimal approach.

| 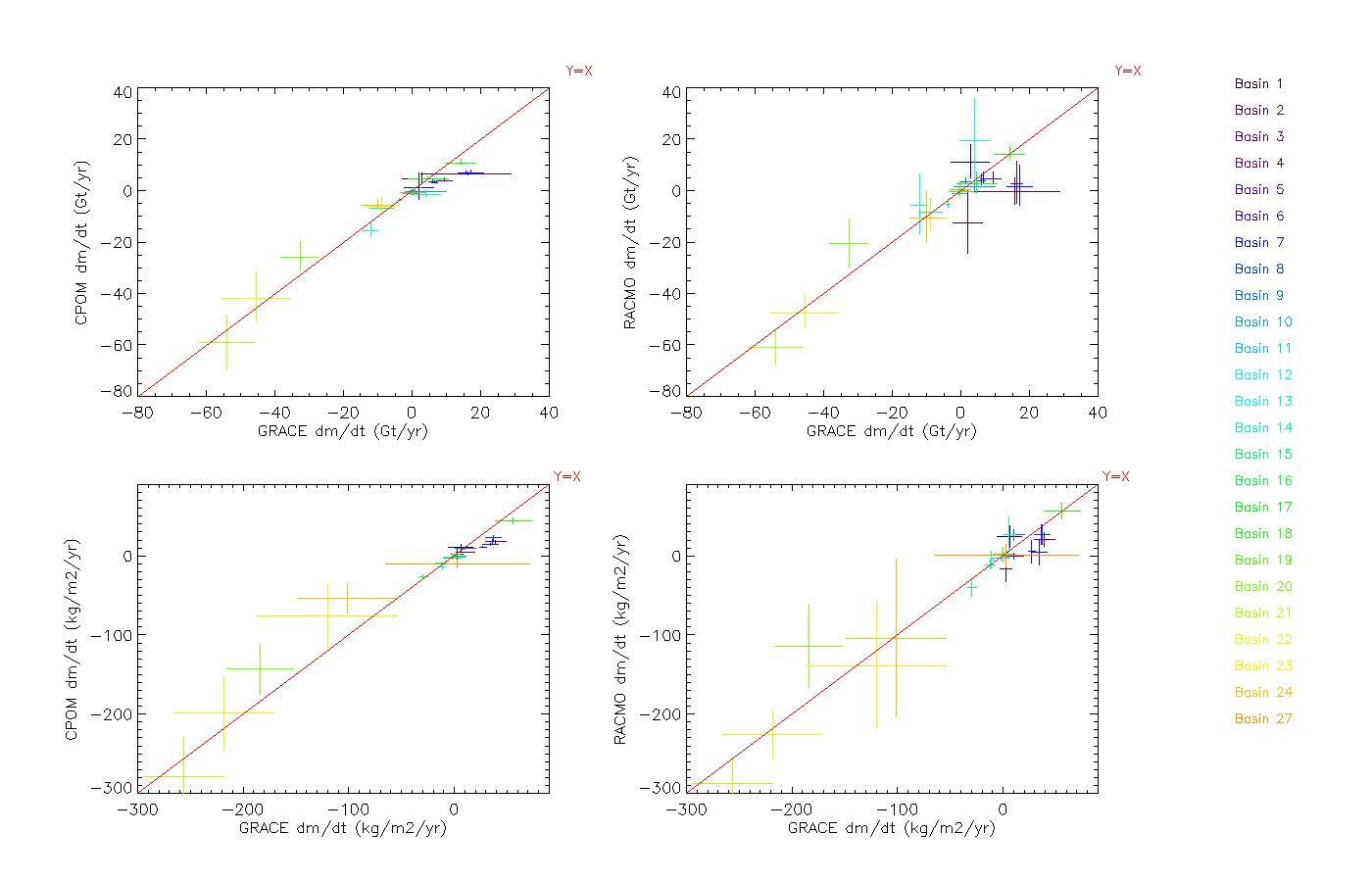 |
| --- |
| **Figure S14.** Scatter plots of (top) mass balance and (bottom) specific mass balance estimates determined within 25 drainage basins of East and West Antarctica and the Antarctic Peninsula from GRACE and from satellite altimetry using the preferred scenario (left) and the alternative scenario (right) employing direct corrections from a firn density model and a regional climate model. |

| **Comparison** | **Parameter** | **Units** | **r^2^** | **RMS** | **s.d.** | **μ** |
| --- | --- | --- | --- | --- | --- | --- |
| Preferred scenario | Mass balance | Gt/yr | 0.97 | 4.5 | 4.4 | -1.5 |
| FDM+RCM scenario | Mass balance | Gt/yr | 0.90 | 7.6 | 7.7 | -1.3 |
| Preferred scenario | Specific mass balance | kg/m^2^/yr | 0.98 | 18.9 | 19.3 | -0.1 |
| FDM+RCM scenario | Specific mass balance | kg/m^2^/yr | 0.97 | 19.9 | 20.2 | -2.4 |
| **Table S4.** Comparison of mass balance and specific mass balance estimates determined within 25 drainage basins of East and West Antarctica and the Antarctic Peninsula from GRACE and from satellite altimetry using the preferred scenario and the alternative scenario employing direct corrections from a firn density model (FDM) and a regional climate model (RCM). Statistics included are the correlation coefficient (r^2^), the root mean square difference (RMS), the standard deviation of the difference (s.d.), and the mean difference (μ). | | | | | | |

**References**

Arthern, R. J. and Wingham, D. J.: The natural fluctuations of firn densification and their effect on the geodetic determination of ice sheet mass balance, Climatic Change, 40, 605-624, 1998.

Bamber, J. L.: Ice-Sheet Altimeter Processing Scheme, International Journal of Remote Sensing, 15, 925-938, 1994.

Boening, C., Lebsock, M., Landerer, F., and Stephens, G.: Snowfall-driven mass change on the East Antarctic ice sheet, Geophysical Research Letters, 39, 2012.

Brown, G. S.: The average impulse response of a rough surface and its applications, IEEE Transactions of Antennas and Propogation, 25, 67-74, 1977.

Cornford, S. L., Martin, D. F., Graves, D. T., Ranken, D. F., Le Brocq, A. M., Gladstone, R. M., Payne, A. J., Ng, E. G., and Lipscomb, W. H.: Adaptive mesh, finite volume modeling of marine ice sheets, J. Comput. Phys., 232, 529-549, 2013.

Davis, C. H.: Comparison of ice-sheet satellite altimeter retracking algorithms, Ieee Transactions on Geoscience and Remote Sensing, 34, 229-236, 1996.

Davis, C. H.: A robust threshold retracking algorithm for measuring ice-sheet surface elevation change from satellite radar altimeters, IEEE Transactions on Geoscience and Remote Sensing, 35, 974-979, 1997.

Davis, C. H. and Ferguson, A. C.: Elevation change of the Antarctic ice sheet, 1995-2000, from ERS-2 satellite radar altimetry, Ieee Transactions on Geoscience and Remote Sensing, 42, 2437-2445, 2004.

Flament, T. and Remy, F.: Antarctica volume change from 10 years of Envisat altimetry, in: International Geoscience and Remote Sensing Symposium (IGARSS)2012, 1848-1851.

Groh, A. and horwath, M.: The method of tailored sensitivity kernels for GRACE mass change estimates, European Geophysical Union, Vienna, 2016.

Helm, V., Humbert, A., and Miller, H.: Elevation and elevation change of Greenland and Antarctica derived from CryoSat-2, Cryosphere, 8, 1539-1559, 2014.

Hogg, A. E., Shepherd, A., Cornford, S. L., Briggs, K. H., Gourmelen, N., Graham, J. A., Joughin, I., Mouginot, J., Nagler, T., Payne, A. J., Rignot, E., and Wuite, J.: Increased ice flow in Western Palmer Land linked to ocean melting, Geophysical Research Letters, 44, 4159-4167, 2017.

Horwath, M., Legrésy, B., Rémy, F., Blarel, F., and Lemoine, J. M.: Consistent patterns of Antarctic ice sheet interannual variations from ENVISAT radar altimetry and GRACE satellite gravimetry, Geophysical Journal International, 189, 863-876, 2012.

Iijima, B. A., Harris, I. L., Ho, C. M., Lindqwister, U. J., Mannucci, A. J., Pi, X., Reyes, M. J., Sparks, L. C., and Wilson, B. D.: Automated daily process for global ionospheric total electron content maps and satellite ocean altimeter ionospheric calibration based on Global Positioning System data, Journal of Atmospheric and Solar-Terrestrial Physics, 61, 1205-1218, 1999.

Ivins, E. R., James, T. S., Wahr, J., O. Schrama, E. J., Landerer, F. W., and Simon, K. M.: Antarctic contribution to sea level rise observed by GRACE with improved GIA correction, J. Geophys. Res. B Solid Earth, 118, 3126-3141, 2013.

Joughin, I., Rignot, E., Rosanova, C. E., Lucchitta, B. K., and Bohlander, J.: Timing of recent accelerations of Pine Island Glacier, Antarctica, Geophysical Research Letters, 30, 1706, 2003.

Joughin, I. and Tulaczyk, S.: Positive mass balance of the Ross Ice Streams, West Antarctica, Science, 295, 476-480, 2002.

Khvorostovsky, K. S.: Merging and analysis of elevation time series over greenland ice sheet from satellite radar altimetry, IEEE Transactions on Geoscience and Remote Sensing, 50, 23-36, 2012.

Konrad, H., Gilbert, L., Cornford, S. L., Payne, A., Hogg, A., Muir, A., and Shepherd, A.: Uneven onset and pace of ice-dynamical imbalance in the Amundsen Sea Embayment, West Antarctica, Geophysical Research Letters, 44, 910-918, 2017.

Li, X., Rignot, E., Morlighem, M., Mouginot, J., and Scheuchl, B.: Grounding line retreat of Totten Glacier, East Antarctica, 1996 to 2013, Geophysical Research Letters, 42, 8049-8056, 2015.

Ligtenberg, S. R. M., Helsen, M. M., and Van Den Broeke, M. R.: An improved semi-empirical model for the densification of Antarctic firn, Cryosphere, 5, 809-819, 2011.

McMillan, M., Shepherd, A., Sundal, A., Briggs, K., Muir, A., Ridout, A., Hogg, A., and Wingham, D.: Increased ice losses from Antarctica detected by CryoSat-2, Geophysical Research Letters, 41, 3899-3905, 2014.

Melchior Van Wessem, J., Jan Van De Berg, W., Noël, B. P. Y., Van Meijgaard, E., Amory, C., Birnbaum, G., Jakobs, C. L., Krüger, K., Lenaerts, J. T. M., Lhermitte, S., Ligtenberg, S. R. M., Medley, B., Reijmer, C. H., Van Tricht, K., Trusel, L. D., Van Ulft, L. H., Wouters, B., Wuite, J., and Van Den Broeke, M. R.: Modelling the climate and surface mass balance of polar ice sheets using RACMO2 - Part 2: Antarctica (1979-2016), Cryosphere, 12, 1479-1498, 2018.

Michel, A., Flament, T., and Rémy, F.: Study of the penetration bias of ENVISAT altimeter observations over Antarctica in comparison to ICESat observations, Remote Sens., 6, 9412-9434, 2014.

Mouginot, J., Rignot, E., and Scheuchl, B.: Sustained increase in ice discharge from the Amundsen Sea Embayment, West Antarctica, from 1973 to 2013, Geophysical Research Letters, 41, 1576-1584, 2014.

Nilsson, J., Gardner, A., Sørensen, L. S., and Forsberg, R.: Improved retrieval of land ice topography from CryoSat-2 data and its impact for volume-change estimation of the Greenland Ice Sheet, Cryosphere, 10, 2953-2969, 2016.

Rignot, E., Bamber, J. L., Van Den Broeke, M. R., Davis, C., Li, Y., Van De Berg, W. J., and Van Meijgaard, E.: Recent Antarctic ice mass loss from radar interferometry and regional climate modelling, Nature Geoscience, 1, 106-110, 2008.

Rignot, E., Casassa, G., Gogineni, P., Krabill, W., Rivera, A., and Thomas, R.: Accelerated ice discharge from the Antarctic Peninsula following the collapse of Larsen B ice shelf, Geophysical Research Letters, 31, art-L18401, 2004.

Rignot, E., Casassa, G., Gogineni, S., Kanagaratnam, P., Krabill, W., Pritchard, H., Rivera, A., Thomas, R., Turner, J., and Vaughan, D.: Recent ice loss from the Fleming and other glaciers, Wordie Bay, West Antarctic Peninsula, Geophysical Research Letters, 32, 1-4, 2005.

Rignot, E., Mouginot, J., and Scheuchl, B.: Ice Flow of the Antarctic Ice Sheet, Science, 333, 1427-1430, 2011.

Scambos, T. A., Bohlander, J. A., Shuman, C. A., and Skvarca, P.: Glacier acceleration and thinning after ice shelf collapse in the Larsen B embayment, Antarctica, Geophysical Research Letters, 31, art-L18402, 2004.

Shepherd, A., Ivins, E., Rignot, E., Smith, B., Van Den Broeke, M., Velicogna, I., Whitehouse, P., Briggs, K., Joughin, I., Krinner, G., Nowicki, S., Payne, T., Scambos, T., Schlegel, N., Geruo, A., Agosta, C., Ahlstrøm, A., Babonis, G., Barletta, V., Blazquez, A., Bonin, J., Csatho, B., Cullather, R., Felikson, D., Fettweis, X., Forsberg, R., Gallee, H., Gardner, A., Gilbert, L., Groh, A., Gunter, B., Hanna, E., Harig, C., Helm, V., Horvath, A., Horwath, M., Khan, S., Kjeldsen, K. K., Konrad, H., Langen, P., Lecavalier, B., Loomis, B., Luthcke, S., McMillan, M., Melini, D., Mernild, S., Mohajerani, Y., Moore, P., Mouginot, J., Moyano, G., Muir, A., Nagler, T., Nield, G., Nilsson, J., Noel, B., Otosaka, I., Pattle, M. E., Peltier, W. R., Pie, N., Rietbroek, R., Rott, H., Sandberg-Sørensen, L., Sasgen, I., Save, H., Scheuchl, B., Schrama, E., Schröder, L., Seo, K. W., Simonsen, S., Slater, T., Spada, G., Sutterley, T., Talpe, M., Tarasov, L., Van De Berg, W. J., Van Der Wal, W., Van Wessem, M., Vishwakarma, B. D., Wiese, D., and Wouters, B.: Mass balance of the Antarctic Ice Sheet from 1992 to 2017, Nature, 558, 219-222, 2018.

Shepherd, A., Ivins, E. R., Geruo, A., Barletta, V. R., Bentley, M. J., Bettadpur, S., Briggs, K. H., Bromwich, D. H., Forsberg, R., Galin, N., Horwath, M., Jacobs, S., Joughin, I., King, M. A., Lenaerts, J. T. M., Li, J., Ligtenberg, S. R. M., Luckman, A., Luthcke, S. B., McMillan, M., Meister, R., Milne, G., Mouginot, J., Muir, A., Nicolas, J. P., Paden, J., Payne, A. J., Pritchard, H., Rignot, E., Rott, H., Sørensen, L. S., Scambos, T. A., Scheuchl, B., Schrama, E. J. O., Smith, B., Sundal, A. V., Van Angelen, J. H., Van De Berg, W. J., Van Den Broeke, M. R., Vaughan, D. G., Velicogna, I., Wahr, J., Whitehouse, P. L., Wingham, D. J., Yi, D., Young, D., and Zwally, H. J.: A reconciled estimate of ice-sheet mass balance, Science, 338, 1183-1189, 2012.

Shepherd, A., Wingham, D. J., and Mansley, J. A. D.: Inland thinning of the Amundsen Sea sector, West Antarctica, Geophysical Research Letters, 29, 2002.

Studinger, M.: IceBridge ATM L4 Surface Elevation Rate of Change, Version 299 1, Antarctica subset. Center, N. S. a. I. D. C. D. A. A. (Ed.), Boulder, Colorado, USA, 2014.

Wingham, D. J., Ridout, A. J., Scharroo, R., Arthern, R. J., and Shum, C. K.: Antarctic elevation change from 1992 to 1996, Science, 282, 456-458, 1998.

Wingham, D. J., Shepherd, A., Muir, A., and Marshall, G. J.: Mass balance of the Antarctic ice sheet, Philosophical Transactions of the Royal Society A-Mathematical Physical and Engineering Sciences, 364, 1627-1635, 2006a.

Wingham, D. J., Siegert, M. J., Shepherd, A., and Muir, A. S.: Rapid discharge connects Antarctic subglacial lakes, Nature, 440, 1033-1036, 2006b.

Wingham, D. J., et al.: CryoSat: A mission to determine the fluctuations in Earth's land and marine ice fields, Adv. Space Res., 37, 841–871, 2006c.

Wingham, D. J., Wallis, D. W., and Shepherd, A.: Spatial and temporal evolution of Pine Island Glacier thinning, 1995-2006, Geophysical Research Letters, 36, 2009.

Wouters, B., Bamber, J. L., Van Den Broeke, M. R., Lenaerts, J. T. M., and Sasgen, I.: Limits in detecting acceleration of ice sheet mass loss due to climate variability, Nature Geoscience, 6, 613-616, 2013.

Zwally, H. J., Brenner, A. C., Major, J. A., Bindschadler, R. A., and Marsh, J. G.: Growth of Greenland ice sheet: Measurement, Science, 246, 1587-1589, 1989.
